# Temporal Patterns in Seawater Quality from Dredging in Tropical Environments

**DOI:** 10.1371/journal.pone.0137112

**Published:** 2015-10-07

**Authors:** Ross Jones, Rebecca Fisher, Clair Stark, Peter Ridd

**Affiliations:** 1 Australian Institute of Marine Science, Perth, Western Australia, Australia; 2 Western Australian Marine Science Institution, Perth, Western Australia, Australia; 3 Intelligent Systems, Information and Modelling, College of Science, Technology and Engineering, Townsville, Queensland, Australia; 4 Oceans Institute, University of Western Australia, Perth, Western Australia; Auckland University of Technology, NEW ZEALAND

## Abstract

Maintenance and capital dredging represents a potential risk to tropical environments, especially in turbidity-sensitive environments such as coral reefs. There is little detailed, published observational time-series data that quantifies how dredging affects seawater quality conditions temporally and spatially. This information is needed to test realistic exposure scenarios to better understand the seawater-quality implications of dredging and ultimately to better predict and manage impacts of future projects. Using data from three recent major capital dredging programs in North Western Australia, the extent and duration of natural (baseline) and dredging-related turbidity events are described over periods ranging from hours to weeks. Very close to dredging i.e. <500 m distance, a characteristic features of these particular case studies was high temporal variability. Over several hours suspended sediment concentrations (SSCs) can range from 100–500 mg L-1. Less turbid conditions (10–80 mg L-1) can persist over several days but over longer periods (weeks to months) averages were <10 mg L-1. During turbidity events all benthic light was sometimes extinguished, even in the shallow reefal environment, however a much more common feature was very low light ‘caliginous’ or daytime twilight periods. Compared to pre-dredging conditions, dredging increased the intensity, duration and frequency of the turbidity events by 10-, 5- and 3-fold respectively (at sites <500 m from dredging). However, when averaged across the entire dredging period of 80–180 weeks, turbidity values only increased by 2–3 fold above pre-dredging levels. Similarly, the upper percentile values (e.g., P99, P95) of seawater quality parameters can be highly elevated over short periods, but converge to values only marginally above baseline states over longer periods. Dredging in these studies altered the overall probability density distribution, increasing the frequency of extreme values. As such, attempts to understand the potential biological impacts must consider impacts across telescoping-time frames and changes to extreme conditions in addition to comparing central tendency (mean/median). An analysis technique to capture the entire range of likely conditions over time-frames from hours to weeks is described using a running means/percentile approach.

## Introduction

Maintenance and capital dredging for ports and coastal infrastructure projects represents a potential risk to tropical marine environments. Dredging the seabed and subsequent dredge-material disposal releases sediment into the seawater column creating plumes that can drift onto nearby benthic habitats. Elevated suspended sediment concentrations (SSCs) can affect filter and suspension feeders by interfering with food collection [1] and the turbid plumes can reduce submarine irradiance, affecting benthic primary producers such as corals seagrasses and macroalgae [2]. Furthermore, sediments in the seawater column can eventually settle out of suspension, potentially smothering benthic and sessile organisms and forcing them to expend energy self-cleaning [1].

Many studies have attempted to quantify the effects of sediment on corals and coral reefs (reviewed in [1–4]) and the risks associated with dredging in coral reef environments have been well known for many years [5,6]. However, observational or time-series data of seawater quality conditions and behaviours during dredging around coral reefs have rarely been collected and described (but see [7,8]). A fundamentally important principle in ecotoxicology and risk assessment is hazard characterisation. Any attempts to relate a change in the biota to changes in environmental conditions needs a detailed understanding of exposure pathways and exposure conditions experienced by wildlife. Harris et al. [9] recently argued that one of the weakest aspects of many ecotoxicological studies is the exposure conditions and emphasised the need to justify the concentrations applied with those measured in the environment.

### Temporal variability in turbidity

SSCs and related turbidity are naturally highly variable, both spatially and temporally, and influenced by a wide range of factors, such as waves, currents and bed type [10–18]. For muddy-bottomed sites on exposed inner-shelves, SSCs can frequently exceed 20 mg L^-1^, and can regularly exceed 100 mg L^-1^ for 2–3 day periods during strong wave events [10]. Similarly, variation in turbidity at inshore coral reefs can also range from 0.1 to >100 NTU over relatively short periods [19], with >20 NTU typically occurring during high wind and wave events, and values greater than 50 NTU occurring during exceptionally high wind and wave events, such as cyclones [12,18,20,21]. Any attempt to characterise the extraordinary conditions and hazards posed by dredging must be carried out in the context of this natural variability, and accordingly, data needs to span a relatively long sample period (typically months). High frequency time series data of turbidity measurement over such long durations are expensive to implement and relatively rare [10].

One of important questions for examining the effects of poor seawater quality associated with dredging on benthic organisms is what the appropriate time frame for analysis is. This question should be framed within the context of the biology of the benthic organisms, the duration of their life-history stages and especially sensitive stages. For example, in corals, the life-cycle consists of multiple stages involving gametogenesis, spawning, fertilisation and embryonic and larval development, and then settlement and metamorphosis to a benthic adult stage. These stages can range from minutes to months and for the adults, years, and each are possibly susceptible to turbidity generation. Thus, an understanding of how seawater quality varies due to dredging (and naturally) across the full range of temporal scales from minutes to months will be required to characterise the hazards posed to corals generally.

Seawater-quality data are usually recorded at relatively fine temporal scales (e.g., minutes, [22]), and aggregated to coarser time scales for the purposes of reporting. The summary statistics used (mean versus median etc), as well as the temporal scale adopted (hours, days, weeks) can dramatically affect the interpretation of the data [10]. Short periods of high SSCs or low light are ecologically significant and the importance of these events are not clear or reflected in median values and especially over longer term averages [23,24]. If the hazards associated with dredging are to be characterised thoroughly, they need to be expressed both with respect to changes in central tendency, but also in terms of changes in upper (e.g., maximum, 95^th^ percentile) and lower bounds.

### Dredging programs in NW Australia

In tropical Australia there has been a recent sequence of major capital dredging campaigns associated with a resources boom and the need for coastal facilities for the export of minerals and petroleum products. Three of the most significant dredging campaigns occurred in the Pilbara region of Western Australia (WA), at the Burrup Peninsula in Dampier Archipelago, and at nearby Barrow Island and Cape Lambert. These projects involved dredging millions of cubic metres of sediment in the nearshore environment to create access channels, turning basins, berth pockets, jetties and material offloading facilities, and the subsequent disposal of the sediment at dredge material placement grounds [25]. The Pilbara projects were all large-scale capital dredging programs with multiple dredges operating nearly continuously (24 h a day for 7 days a week) and over extended periods. They were significant by global standards and occurred in sensitive tropical marine environments containing coral reefs and other benthic primary producer habitats [26]. The projects also occurred in three very different marine settings representing the range of environments that corals occupy in tropical Australia and elsewhere in the world: an offshore, ‘clear seawater’ environment (Barrow Island), an exposed nearshore cape or headland (Cape Lambert), and an enclosed inshore turbid reef environment (Mermaid Sound, Burrup Peninsula) of the Dampier Archipelago.

The state and federal regulatory conditions for the Pilbara dredging projects required detailed seawater quality monitoring programs involving measurements of turbidity and light levels on sub-hourly time scales at multiple reference and potential impact sites. Measurements were made at different distances from the dredging and over extended periods (months to years), and in some cases included extended pre-dredging baseline periods [25]. Data from these studies have been made available by the dredging proponents for scientific study, providing a unique opportunity to explore, for the first time, the impacts that dredging has on seawater quality in reef areas across broad temporal scales. These data include extensive baseline time series (in some cases), and thus allow the characterisation of the effects on seawater quality caused by dredging in the context of inherent natural variability.

The aim of this study is to thoroughly characterise the hazard caused by dredging activities altering seawater quality in reefal environments. We describe the conditions reef communities may encounter *in situ* as a result of dredging, including the nature and duration of episodic high SSC and low light ‘turbidity events’ and how the nature of these events varies over periods of time from minutes and hours to weeks and months. The results are valuable for future experiments and the design of more environmentally realistic laboratory-based, *ex situ* studies of the effects of turbidity and light on reef biota such as filter feeders (i.e. sponges and ascidians), fish, corals and other primary producers (i.e. seagrasses). Together with analyses of spatial patterns (i.e. distance from dredging) of seawater quality, and effects of the dredging projects on the underlying reef communities (both of which will be published elsewhere) the data are important for developing seawater quality thresholds for dredging programs to improve the ability to predict and manage the impact of future dredging projects.

## Materials and Methods

Turbidity is a measure of light scattering caused mainly by suspended sediment, algae, micro-organisms and other particulate matter [10,18] and in the seawater column is conventionally measured using a nephelometer as Nephelometric Turbidity Units (NTU). Turbidity is a function of suspended sediment concentrations although conversion between turbidity and SSC varies in response to a wide range of sediment characteristics, particularly those related to grain size and type, which also change with time [27]. In general SSC can be related to turbidity by a linear relationship with a conversion factor of between 1 and 4 [10].

**Fig 1:**
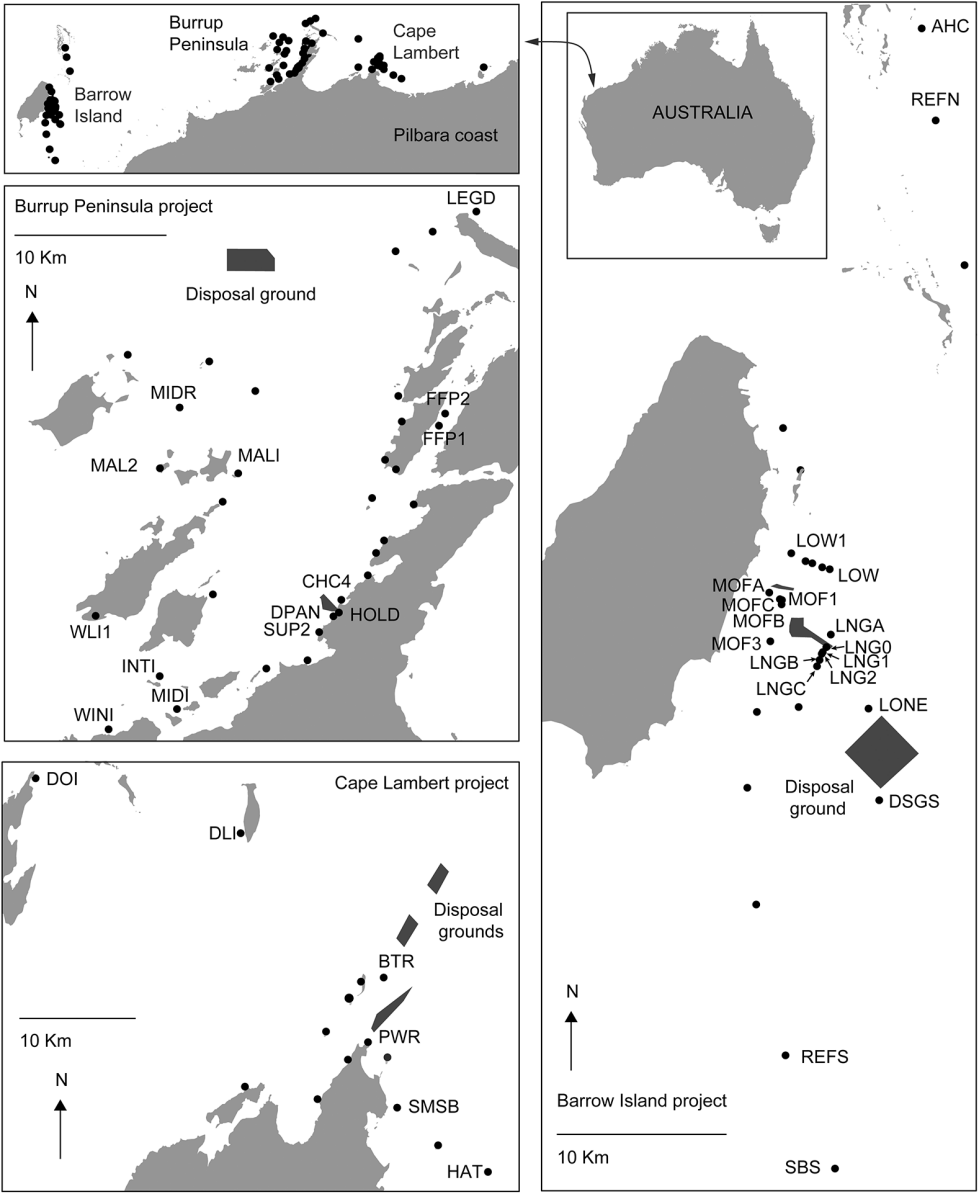
Seawater quality monitoring and reference (Ref.) sites for the Barrow Island (MS800), Burrup Peninsula (MS757), and Cape Lambert (MS840) dredging projects in the Pilbara region (Western Australia). Only sites that were near (<2 km) from the primary dredging activity and those that were considered un-impacted by dredging (references sites) were used in the analyses here and are labelled. Detail site information can be found in the (Table 1 and S1 File). The ministerial approval statements (MS) for these projects are available on the WA EPA website: http://www.epa.wa.gov.au. Dredge material placement sites (spoil grounds) and primary excavation areas are indicated as dark shaded boxes.

Seawater quality data (turbidity) were collected at 32 sites for the Burrup Peninsula Project, 26 sites during the Barrow Island project, and 15 sites at the Cape Lambert project (Fig 1, Table 1). Many of these sites included baseline periods before dredging started with some baseline periods covering up to 786 days. Seawater quality data for these projects were collected using instruments mounted ~40 cm from the seabed on steel framed *in situ* monitoring platforms. The instruments used and logging and download frequencies for each project varied (see Table 1).

**Table 1:** Data type collected and instruments used across three major dredging projects in the Pilbara (Western Australia) since 2007 including start and finish dates and volumes dredged.

**Burrup Peninsula (MS757)**	
Project works	Capital dredging project to create a navigation channel (16 km, 12.5 m seawater depth), turning basin (600 m radius, 12.5 m seawater depth), and berth pocket (400 m × 60 m, 13.5 m seawater depth)
Volume dredged	~12.5 Mm^3^
Dredging Period (d)	22 Nov 2007 to 21 May 2010 (911 days). Baseline days: Turbidity 5–123 (15) NTU, Light: 0–117(109) μmol photons m^-2^ s^-1.^ Dredging days: Turbidity 47–984 (905) NTU, Light 0–82 (82) μmol photons m^-2^ s^-1^
Instrumentation	(1) Optical backscatter (OBS) (JCU Geo-physical Lab), (2) Wetlabs (ECO-NTU-SB OBS turbidity recorder), (3) Alec Instruments (COMPACT CLW—Miniature Turbidity/Chlorophyll Data Logger) (HOLD and DPAN only). Readings every 30 minutes. 32 sites in total (S1 File).
Sediment type:	Surficial sediments are mixed siliciclastic and carbonate unconsolidated sediments ranging from gravel to fine silts. For the nearshore sites, close to the dredging activities, surficial sediments were finer (sand, silt and clay = ~30%) and coarser (sand = 70%, silt 10%, clay 10%) at the more offshore sites. For the nearshore sites (DPAN, HOLD, CHC4) the SSC = Turbidity × 1.174.
**Barrow Island (MS800)**	
Project works	Capital dredging project to create a materials offloading facility (MOF) approach channel (1.6 km, 6.5 m seawater depth), Berthing Pocket dredged to approximately 8 m seawater depth. LNG Jetty access channel and turning basin (900 m circle, 13.5 m seawater depth). LNG berthing Pocket dredged to approximately 15 m seawater depth.
Volume dredged	~7.6 Mm^3^
Dredging Period (d)	19 May 2010 to 31 Oct 2011 (530 days). Baseline days: Turbidity 2–786 (184) NTU, Light: 10–735 (241) μmol photons m^-2^ s^-1^.Dredging days: Turbidity 361–566 (482) NTU, Light 388–548 (474) μmol photons m^-2^ s^-1^
Instrumentation:	Sideways mounted optical backscatter device (nephelometer) and Photosynthetically Active Radiation (PAR) was recorded using a 2π quantum sensor (JCU Geo-physical Lab, see Thomas & Ridd 2005). Readings every 10 minutes. 36 sites in total (S1 File)
Sediment type:	Predominantly unconsolidated, undisturbed carbonate sediments forming a thin veneer (0.5–3 m thick) overlying limestone pavements ranging from rubble to typically gravelly sand mixed with fine silts and clays. Low TOC content <0.8%. Sediments at deeper sites were typically finer. SSC = Turbidity × range of 1.1 to1.6
**Cape Lambert (MS840)**	
Project works	Capital dredging project to create an approach area and channel (15.6 m seawater depth), turning basin (10.0 m seawater depth) and berth pocket (20 m seawater depth), and tug harbour extension (6.8 m seawater depth)
Volume dredged	~14 Mm^3^
Dredging Period (d)	22 Dec 2010 to 15 Sept 2012 (633 days). Baseline days: Turbidity 13–536 (399) NTU, Light: 0–279 (91) μmol photons m^-2^ s^-1^.Dredging days: Turbidity 629–699 (685) NTU, Light 0–686 (649) μmol photons m^-2^s^-1^
Instrumentation:	(1) Wetlabs (ECO-NTU-SB OBS turbidity recorder) every 30 mins. ALEC ALW-CMP loggers. (2) WET Labs ECO-PAR-SB (30 min) ALEC ALW-CMP. Readings every 30 minutes. 15 sites in total (S1 File))
Sediment type:	Unconsolidated predominantly carbonate sediments, composed of medium to coarse sand (70–90%) at a range of 1–5 km from dredging but typically finer sediments (fine sands, silt and clay) closer to the nearshore areas. SSC = A[Turbidity] e^B[Turbidity]D^ +C, where A = 0.670 B = 0.256, C = 0.275 and D = 0.0391

The range in number of seawater quality sample days during baseline (Baseline days) and dredging (Dredging days) are included at each location for turbidity and light data. Values in parentheses represent the median number of sampling days across sites where that seawater quality parameter was measured. MS refers to the Federal Ministerial approval Statement, searchable on the WA EPA website: http://www.epa.wa.gov.au).

As the primary purpose of this paper was to describe the seawater quality characteristics in the immediate vicinity of dredging activity, we have limited the analysis for each of the projects to those monitoring sites <2 km from the primary area of dredging activity, and those sites that were considered to be un-impacted by the dredging activity (reference sites, see labelled sites, Fig 1). Full details for each site in the present analysis, including total baseline and dredge period sampling days, seawater depths (where available) and distances of the monitoring sites from the main dredging activities are listed in Table 1 and the supplementary data (S1 File).

All seawater quality data provided by the proponents of the various projects were processed similarly to ensure data integrity and remove potentially erroneous values (see below) and time standardised to decimal Julian days, where the start of dredging was used as the origin. This ensures that negative values of Julian day represent the baseline period, and positive values represent days during the dredging program. For all turbidity data, any values <0 NTU were removed, and a smoothing filter was applied where for any value >3 NTU, if the value was more than 2.5× the mean of the preceding and following value, it was replaced with the mean of the two values. This smoothing filter was initially applied to reduce any high single point anomalies that may be due to material or organisms (e.g., fish or algae) passing in front of the sensor at the time the reading was taken. For both the turbidity and light datasets for each location, raw data were plotted as time series and inspected visually for anomalies and any evidence of logger or wiper failure. Suspect data points and/or sections were identified in a data cleaning log which was subsequently used to screen out this data for all analyses. A range of different types of anomalies were removed and included: erratic spikes or peaks representing large changes in turbidity lasting for short periods of time that could not possible be due to natural (or dredge induced) changes in turbidity and/or were not reflected in changes in light data (where this was also available); sections of systematically fluctuating turbidity patterns occurring on the same period as the logger wipers (very likely due to logger error, only removed when these caused extreme fluctuations in turbidity readings); sudden elevations or drops in turbidity readings (occurring suddenly over the time of a single reading, rather than rising across several readings as would be expected by natural turbidity patterns) that indicate an issue with sensor calibration; other sensor ‘drift’ issues where there was a pattern of increasing turbidity and a sudden drop over the space of a single reading, indicating a sensor drift and re-calibration issue. For the turbidity data from the Burrup Peninsula project there was an issue with data obtained immediately after the commencement of dredging for the HOLD and DPAN sites (Fig 1), where there were clear periods of instrumental ‘drift’. Because these sites are very close to the dredging (0.32 and 0.56 km for the HOLD and DPAN sites, respectively) during the relevant period they are of particular value in characterising the near dredge seawater quality conditions. Rather than exclude this data entirely (as was done for other sections of data from the three projects when there were plenty of other representative sites available), these data were instead adjusted assuming linear drift of the sensors across the time period. While this assumption of linear drift might introduce some small error, given the value of this data and the large values of turbidity that occurred during this time, it is unlikely this assumption would impact on the outcomes of the analysis.

For the light measurements, any night-time data collected one hour before predicted sunrise or one hour after predicted sunset, and any values <0 and >2000 μmol photons m^-2^ s^-1^ were removed. Sunrise and sunset estimates were obtained and applied at monthly intervals.

All turbidity data were aggregated for all sites and retained at the finest temporal resolution (10 or 30 min, depending on the logger type and dredging project (Table 1) or aggregated to a daily mean or percentile value as required for various analyses). Light data at the finest temporal resolution were fitted using a Generalised Additive Model (GAM) for each day separately using the mgcv package [28] in R [29]. Days for which insufficient light data were available throughout the full daylight cycle were removed and not included in the analysis. Each fitted daily model was then used to estimate photosynthetically active radiation (PAR, 400–750 nm) values for every second throughout the daylight period, based on monthly sunset and sunrise times. The sum of the per second quantum flux measurements were then added together to calculate the daily light integral (DLI) as mol photons m^-2^ d^-1^.

### Time series and probability profiles

To examine the overall impacts that dredging has on turbidity and irradiance, representative dredge impact and non-impacted (reference) sites were selected across the three projects and used to explore changes in the time series between the baseline and dredging periods. Representative ‘near’ dredge sites were selected as those closest (<2 km) to the primary dredging activity displaying the longest and most continuous time series throughout the baseline and dredge periods. Similarly, representative ‘reference’ sites were selected as those within the set of sites considered to be un-impacted by dredging activities due to their greater distance from the dredging activity and displaying the longest and most continuous time series throughout the baseline and dredge periods (<6% of days missing throughout the dredging phase). Although only one or two representative sites are shown here, plots for all ‘near’ dredge and reference sites are included in the online supplementary information (Figures A, B, C in S2 File).

While characterisation for turbidity was possible across all three dredging programs, analyses based on light data have only been included for the Barrow Island program, as data for light were either sparse or non-existent during the baseline period or during dredging (or both) for the other programs.

### Intensity (I), Duration (D), Frequency (F) analysis

Turbidity data were used to carry out an intensity, duration and frequency analysis (IDF, see [30]), at both the daily and hourly temporal scales. The approach expands the recognition that it is suspended sediment concentrations and also duration of exposure that causes effects (see [31,32]). In this analysis the data are first aggregated to the appropriate temporal scale by calculating the maximum hourly or daily turbidity values for each dataset for the baseline and during dredge periods. The intensity threshold is then calculated as the 95^th^ percentile of the baseline period (and compared to the 95^th^ percentile of the dredging period). The duration of events where this 95^th^ percentile baseline threshold is exceeded is then determined, and the 95^th^ percentiles of the duration events are then calculated for the baseline period and compared to the 95^th^ percentile of the dredging period. Finally, the frequency with which the 95^th^ percentile duration events for the baseline state were exceeded was also recorded for the baseline periods and periods during dredging.

### Temporal analysis

To examine how the extremes of seawater quality conditions are altered by dredging across a range of time scales, percentile plots of different running mean periods were created for both turbidity and light (where available). Running means of the 10 min or 30 min turbidity/light data were calculated with periods ranging from one hour (for turbidity) or one day (for PAR) to 30 d. Each running time period calculated the average of the previous *N*_*T*_ data points, where *N*_*T*_ is the number of samples in the *T* hour mean. For example, for the two hour running mean (*T* = 2), *N*_*T*_ = 12 as there are six ten-minute samples per hour. The *T* hour running mean at a point in time *t*

$${\bar{x}}_{T}(t)=\frac{1}{N_{T}}\sum_{i=1}^{N_{T}}\;x_{i}\;\left(t\right)$$ (1)

where ${\bar{\text{x}}}_{\text{T})}\left(\text{t}\right)$ is the mean calculated over the previous *T* hours of the data from time *t-T* to time *t* hours, and *x*_*i*_ *(t)* are the *N*_*T*_ data points up to and including time *t*. To avoid biased averages, no ${\bar{x}}_{T}$ value was recorded if more than 20% of the data points for any particular running mean time period calculation were missing. Percentile values of the running mean values ${\bar{\text{x}}}_{\text{T})}\left(\text{t}\right)$ for each running mean period were then calculated. This was done for the pre-dredge and dredge periods.

In R, running means were calculated by converting the data series for each site into an S3 time series object using the zoo function from the zoo library [33] then applying the runmean function from the caTools library [34]. Once running means for each time span were calculated, these were summarised using an average along with various percentile values (50^th^, 80^th^, 99^th^ and 100^th^ [maximum] for turbidity and 50^th^, 20^th^, 5^th^, 1^st^ and 0^th^ [minimum] for PAR). These were plotted as a function of the running mean time span and compared for the pre-dredging and dredging periods.

### Low light periods

High SSCs frequently cause darkness and also very low light or ‘caliginous’ periods reducing underwater irradiances to very low daytime similar to ‘twilight’. The frequency of these low light periods was examined using four different DLI cut-off values, which are equivalent to 12 h of continuous light at instantaneous levels of 20, 10, 5 and 1 μmol photons m^-2^ s^-1^. The latter cut-off value is the precision of the light sensors. Equivalent DLI thresholds based on these per second quantum flux thresholds were determined by summing these for every second across the daylight period, and equate to 0.8, 0.4, 0.2 and 0.04 mol photons m^-2^ d^-1^. Using these thresholds (cut-off values), the total number of days in low light was calculated and normalised per year for each study, for the baseline and dredge periods separately. In addition, the mean number of days in low light per fortnight, as well as the number of consecutive days in low light (summarised as a mean, 80^th^ percentile and maximum) were calculated. For the purposes of calculating continuous days in low light, single missing days of light data were treated as follows: (1) if both the preceding day and following day were defined as low light it was assumed the missing day was also the same; (2) if both the preceding day and following day were defined as ‘light’, it was assumed the missing day was also defined as ‘light’; and, (3) where the preceding day and following day fell into different states the missing day was discarded. This was done to avoid falsely truncating consecutive day calculations where single missing days occurred in the data series. In addition to the proportion of days in low light, the proportion within each day that fell within the low light threshold (i.e. the proportion of the day below the threshold value) was also examined.

## Results

Mean turbidity was low across the 100s of days of the baseline and dredging periods for all three of the major dredging projects (Table 2). Highest baseline turbidity values occurred for the Cape Lambert project (4 NTU) with the Barrow Island and Burrup Peninsula projects showing substantially lower levels (1 and 2 NTU respectively, Table 2). Across site means increased only slightly during the dredging to 3 NTU for the Barrow Island project and 5 NTU for the Cape Lambert and Burrup Peninsula projects (Table 2). Within-site means varied more broadly, with values as high as 7–9 NTU at some sites during dredging at Barrow Island and Cape Lambert (Table 2) and 35 NTU during dredging at one site at Burrup Peninsula (Table 2). Exceptionally high mean values occurred for sites CHC4, DPAN and HOLD for Burrup Peninsula and occurred because these three sites were based on a short data series (~ 3 months in late 2007 and early 2008) collected only during a small window of high dredging activity (see S1 File). For sites surveyed throughout the entire dredging phase in the Burrup Peninsula project, average turbidity values near the dredge site were in the order of 4 NTU, slightly above the precision of the instrumentation (1 NTU).

**Table 2:** Mean turbidity and photosynthetically active radiation (PAR) for the Barrow Island, Cape Lambert and Burrup Peninsula dredging programs.

	Turbidity (NTU)		PAR (μmol photons m^-2^ s^-1^)	
Program	Baseline	Dredging	Baseline	Dredging
Barrow Island	1 (1–3) N = 18	3 (1–7) N = 18	102 (49–320), N = 18	86 (20–288) N = 18
Cape Lambert	4 (1–10) N = 5	5 (2–9) N = 5		
Burrup Peninsula	2 (0–3) N = 11	5 (1–35) N = 11		

Values are the mean across all sites, with values in parentheses showing the range of within site means at each location. N indicates that number of sites used for each location

### Time series and probability profiles

**Fig 2:**
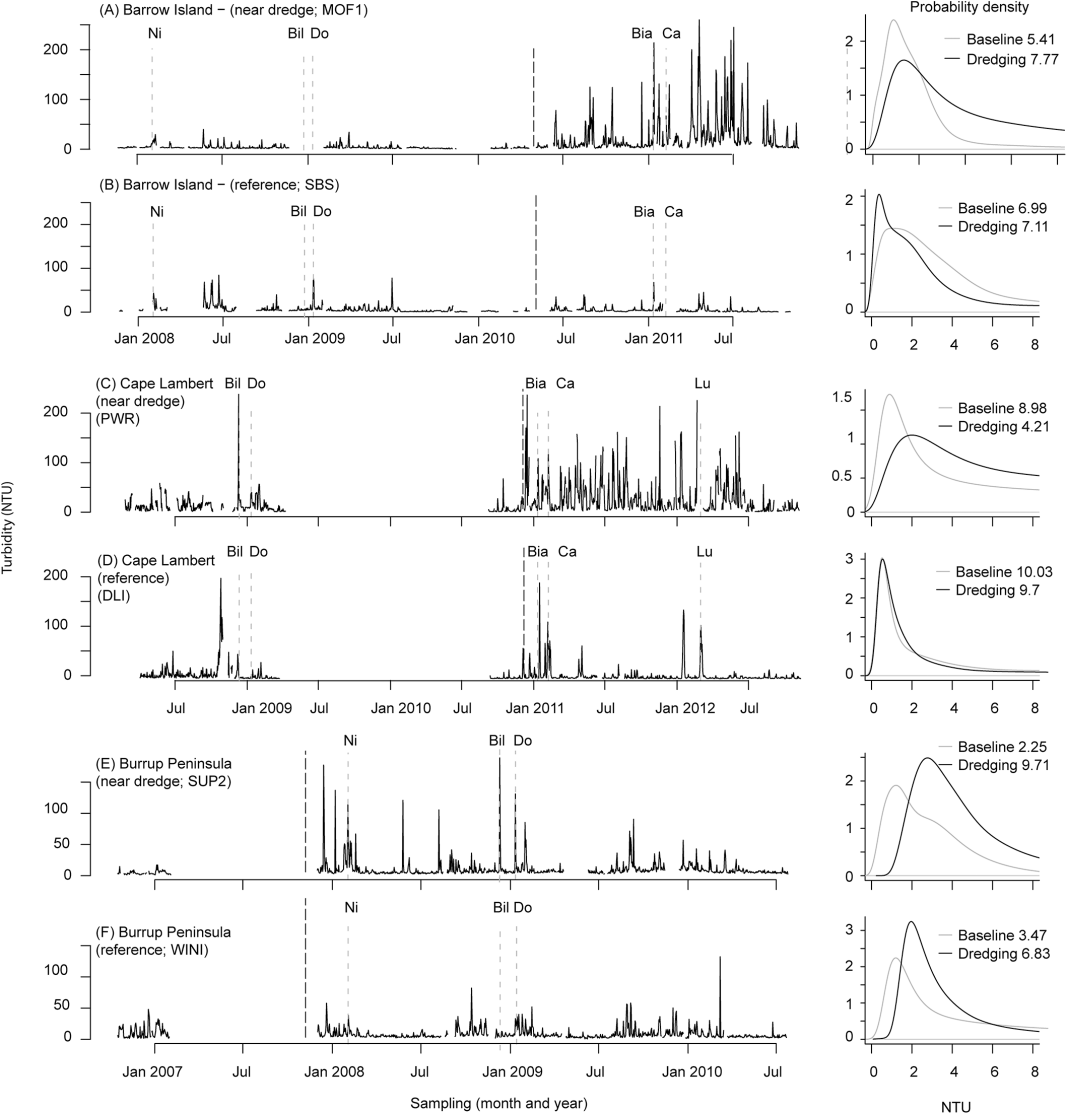
Instantaneous turbidity as maximum daily NTU (left column) and probability density function (far-right panels) at (A) MOF1 (B), SBS during the Barrow Island dredging project, (C) PWR and (D) DLI during the Cape Lambert dredging project, and (E) SUP2 and (F) WINI during the Burrup Peninsula project. LNGI, PWR and SUP2 represent dredge impacted sites whereas SBS, DLI and WINI represent sites un-impacted by dredging (reference sites). The thick solid line on the left hand plots indicates the start of dredging for each project, whereas the dashed lines indicate the timing of cyclone events that may have had the potential to cause sustained periods of very rough seas in this region (Puotinen, pers comm) based on the cyclone size, intensity and proximity to sites (Beeden et al 2015). Annotations under each axis indicate each cyclone event, as follows: Nicholas (N), category 4; Billy (Bil), category 3; Dominic (Do), category 2; Bianca (Bia) category 4; Carlos (Ca), category 3; Lua (Lu) category 4. Cyclone categories indicate the intensity (Australian Ranking Scale) of each cyclone at closest approach to the sites. Time series and probability density function plots for all sites for the three projects can be found in the online supplementary information (Figures, A, B, C in S2 File).

Turbidity was variable over time at all three locations, characterised by sudden peaks that occurred occasionally during the baseline period and more frequently throughout the dredging phase for each project (Fig 2A). While the baseline period was more stable (no peaks >50 NTU) during the Barrow Island dredging project (Fig 2A and 2B), peaks of >100 NTU for ~2 days occurred during the baseline of the Cape Lambert project at both impact (Fig 2C) and reference (Fig 2D) locations. These large peaks in turbidity did not appear to be associated with a known cyclonic event (Fig 2C and 2D). The baseline data time series for the Burrup Peninsula project were substantially shorter than for the other two projects but the available data did not tend to show elevated peaks in turbidity (Fig 2E and 2F). Despite variation among the three projects in the baseline turbidity profiles, representative dredge impacted sites clearly show a much greater frequency of high turbidity peaks (>50–100 NTU) in addition to those associated with cyclone activity during the dredging phase compared to the baseline period for all three locations (Fig 2A, 2C and 2E).

Probability density profile plots for the representative impact and reference locations clearly show an upward shift in the turbidity profile during the dredging period relative to baseline, such that there is a decrease in the skewness, but only at impact locations (Fig 2).

### Temporal scales analysis

**Fig 3:**
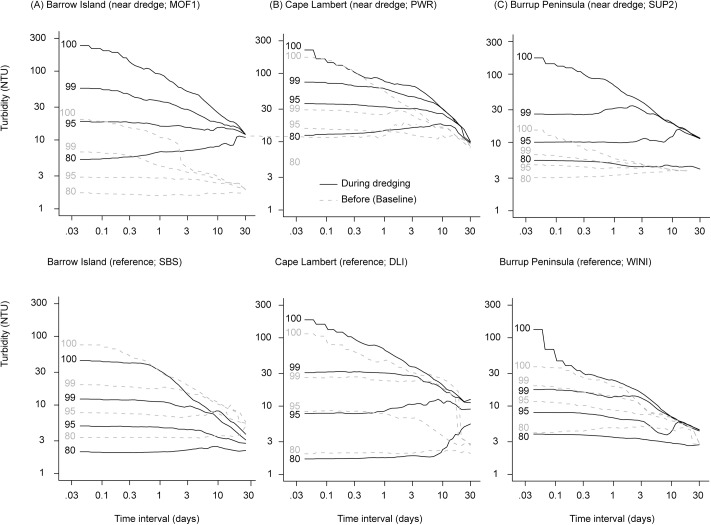
Running means percentile analysis for turbidity (NT U) at sites close to dredging (<2 km) or at reference sites during the Barrow Island, Cape Lambert and Burrup Peninsula dredging projects (see Fig 1). The 100^th^ (maximum), 99^th^ and 95^th^ and 80^th^ percentiles for the running mean turbidity are shown. Percentiles were calculated separately for the baseline period (dashed grey lines) and during dredging (black solid lines).

To illustrate how the temporal scale influences the measureable scale of impact that dredging has on the seawater quality, running means analysis was used over multiple time frames from hours to up to 30 d. The full output is presented in the online supporting information (Figs A-D in S2 File) and representative figures are shown here for turbidity (Fig 3).

Running mean profiles show similar patterns across all three projects, with upper percentile values of turbidity (100^th^, 99^th^ and 95^th^) generally decreasing as temporal scale is increased from hours to weeks (Fig 3). Values for the 80^th^ and 50^th^ were relatively stable across the various time scales examined here (Fig 3).

For the Barrow Island project the SBS reference site located 30 km from the dredging activity (Fig 1) has running mean turbidity values across time frames from hours to weeks that only differed slightly between the baseline period and during the dredging program (i.e. the dotted lines and solid lines largely overlap, Fig 3B). In contrast, at the MOF1 site (located ~0.5 km from the dredging, Fig 1), turbidity levels during the dredging program over one hour, one day and one week time periods were at least an order of magnitude higher than during the baseline period (Fig 3A).

The dramatic shift in seawater quality between the baseline and dredging periods was also seen at the representative sites closest to dredging during the Cape Lambert and Burrup Peninsula projects (Fig 3B and 3C). However, due to occasional periodic peaks in turbidity during the baseline period for the Cape Lambert project, the separation between baseline and dredge periods was slightly less pronounced at this location for the extreme upper percentiles over shorter time frames (Fig 3B).

**Fig 4:**
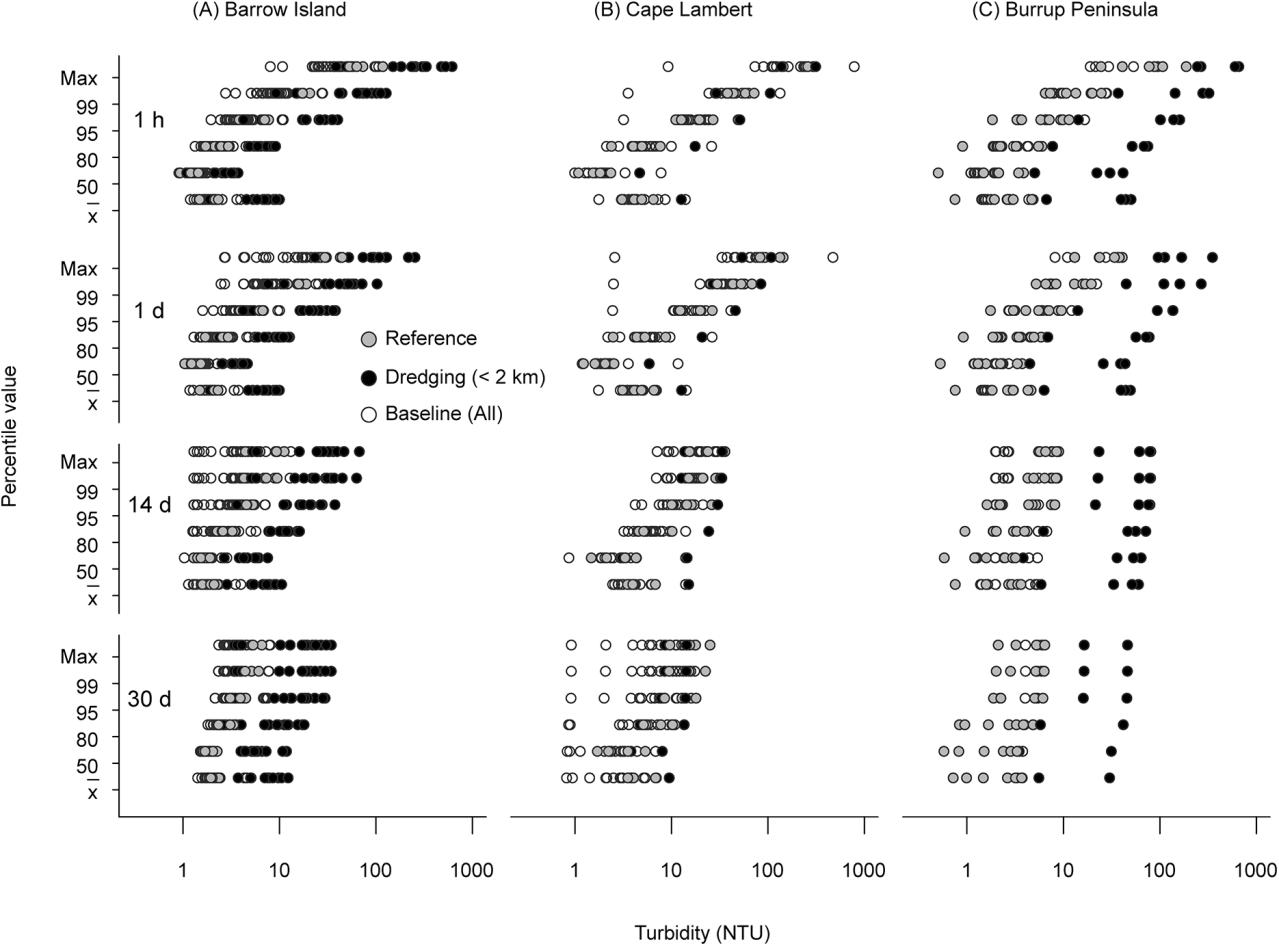
Turbidity (NTU) percentile values for running means calculated on time scales of one hour (h) and 1, 14 and 30 days (d) for all sites at (A) Barrow Island, (B) Cape Lambert and (C) Burrup Peninsula dredging projects. White symbols represent percentiles for the baseline period (pre-dredging period), grey symbols represent reference sites during the dredging period and black symbols represent sites close to (<2 km) the dredging.

Examined collectively across all locations, the upwards shift in running mean turbidity (Fig 4), and downwards shift in available light (see below) at both fine (daily) and coarse (fortnightly, monthly) temporal scales is clearly evident at many near dredge locations for the Barrow Island Project (Fig 3A). Lower percentile values (50^th^, 80^th^) tend to show some overlap between dredge impacted sites during dredging and those occurring for reference sites and baseline periods, with values of ~1 NTU dominant across all time frames (Figs 3A and 4A, Table 3). Upper bounds (95^th^ to 100^th^ percentile values) however, show a marked increase for sites near (<2 km) dredging activity, with hourly means maxima of ~300 NTU and 30 d running means of ~30 NTU (Figs 3B and 4B, Table 3).

**Table 3:** Turbidity (NTU) percentile values for various running mean time periods for the Barrow Island, Cape Lambert and Burrup Peninsula dredging projects.

		*P*_100_ (max)	*P*_99_	*P*_95_	*P*_80_	*P*_50_	Mean
Barrow Island project Baseline/Reference							
	1 h	29, 35, 6–104	7, 8, 2–32	3, 4, 1–13	2, 2, 1–4	1, 1, 1–2	1, 1, 1–3
	1 d	12, 14, 2–61	5, 7, 2–28	3, 3, 1–12	2, 2, 1–4	1, 1, 1–2	1, 1, 1–3
	14 d	3, 4, 1–18	3, 4, 1–16	2, 3, 1–10	2, 2, 1–4	1, 1, 1–2	1, 1, 1–3
	30 d	2, 3, 2–10	2, 3, 2–10	2, 3, 2–10	2, 2, 1–5	1, 1, 1–3	1, 2, 1–3
Barrow Island project Dredging period							
	1 h	224, 233, 106–434	49, 51, 24–90	19, 19, 11–28	6, 6, 3–8	2, 3, 2–5	5, 5, 3–7
	1 d	67, 77, 33–179	36, 37, 18–72	18, 18, 9–27	7, 7, 4–9	3, 3, 2–6	5, 5, 3–7
	14 d	19, 20, 4–47	16, 18, 4–44	12, 13, 4–26	8, 8, 2–11	4, 4, 2–7	5, 5, 2–8
	30 d	13, 13, 3–24	12, 13, 3–24	9, 11, 3–21	8, 8, 3–13	4, 5, 3–8	5, 6, 3–9
Cape Lambert project Baseline/Reference							
	1 h	154, 149, 7–553	28, 32, 3–94	11, 13, 2–35	4, 5, 1–18	1, 2, 1–5	3, 4, 1–10
	1 d	46, 63, 2–333	25, 26, 2–48	10, 12, 2–29	4, 5, 2–19	1, 2, 1–8	3, 3, 1–10
	14 d	14, 14, 5–25	13, 13, 5–23	8, 9, 3–18	4, 5, 2–10	2, 2, 1–10	3, 3, 2–10
	30 d	7, 7, 1–18	7, 7, 1–16	6, 6, 1–13	4, 4, 1–8	2, 2, 1–5	2, 3, 1–5
Cape Lambert project Dredging period							
	1 h	159, 159, 97–220	48, 48, 21–75	23, 23, 9–36	8, 8, 4–12	2, 2, 2–3	6, 6, 3–9
	1 d	57, 57, 38–76	39, 39, 19–60	21, 21, 9–32	9, 9, 4–15	3, 3, 2–4	6, 6, 3–9
	14 d	17, 17, 10–24	16, 16, 9–23	14, 14, 7–21	10, 10, 4–17	6, 6, 2–10	7, 7, 3–11
	30 d	8, 8, 6–10	8, 8, 6–10	8, 8, 6–10	6, 6, 3–10	4, 4, 3–6	5, 5, 3–7
Burrup Peninsula project Baseline/Reference							
	1 h	15, 28, 13–132	5, 8, 5–20	3, 4, 1–12	2, 2, 1–4	1, 1, 0–3	1, 2, 1–3
	1 d	8, 11, 6–29	5, 7, 4–16	2, 3, 1–9	1, 2, 1–5	1, 1, 0–3	1, 2, 1–3
	14 d	2, 3, 1–6	2, 3, 1–6	2, 3, 1–6	1, 2, 1–5	1, 2, 0–4	1, 2, 1–4
	30 d	4, 3, 1–5	4, 3, 1–4	3, 3, 1–4	2, 2, 1–3	2, 2, 0–3	2, 2, 1–3
Burrup Peninsula project Dredging period							
	1 h	306, 312, 173–463	149, 138, 26–227	85, 73, 10–113	42, 36, 5–53	19, 17, 4–29	30, 25, 5–35
	1 d	99, 128, 68–247	95, 103, 32–189	81, 67, 10–96	45, 38, 5–55	23, 20, 3–31	30, 25, 4–35
	14 d	49, 43, 16–57	49, 43, 16–57	48, 42, 15–56	36, 32, 4–50	31, 28, 3–45	30, 27, 4–42
	30 d	22, 22, 12–33	22, 22, 12–32	22, 22, 11–32	17, 17, 4–29	12, 12, 2–22	13, 13, 4–21

Percentiles were calculated separately for the baseline and reference site data (combined) and for near dredge sites (<2 km) during dredging. Shown are the median, mean and range (min–max) for the 100^th^ (maximum), 99^th^, 95^th^, 80^th^, 50^th^ (median) percentiles and mean for one hour, one day, 14 d and 30 d running mean periods.

The overall patterns were similar for the Burrup Peninsula project (Figs 3C and 4C, Table 3), with turbidity values ranging from ~1 NTU for baseline periods and reference sites up to 30 NTU at the monthly scale, and >300 NTU for maximum hourly running means (Fig 3C, Table 3). The results for the Cape Lambert project were mixed, with turbidity values for baseline and reference sites exceeding the near dredge sites in terms of maximum observed values in some cases (Fig 3C, Table 3).

**Fig 5:**
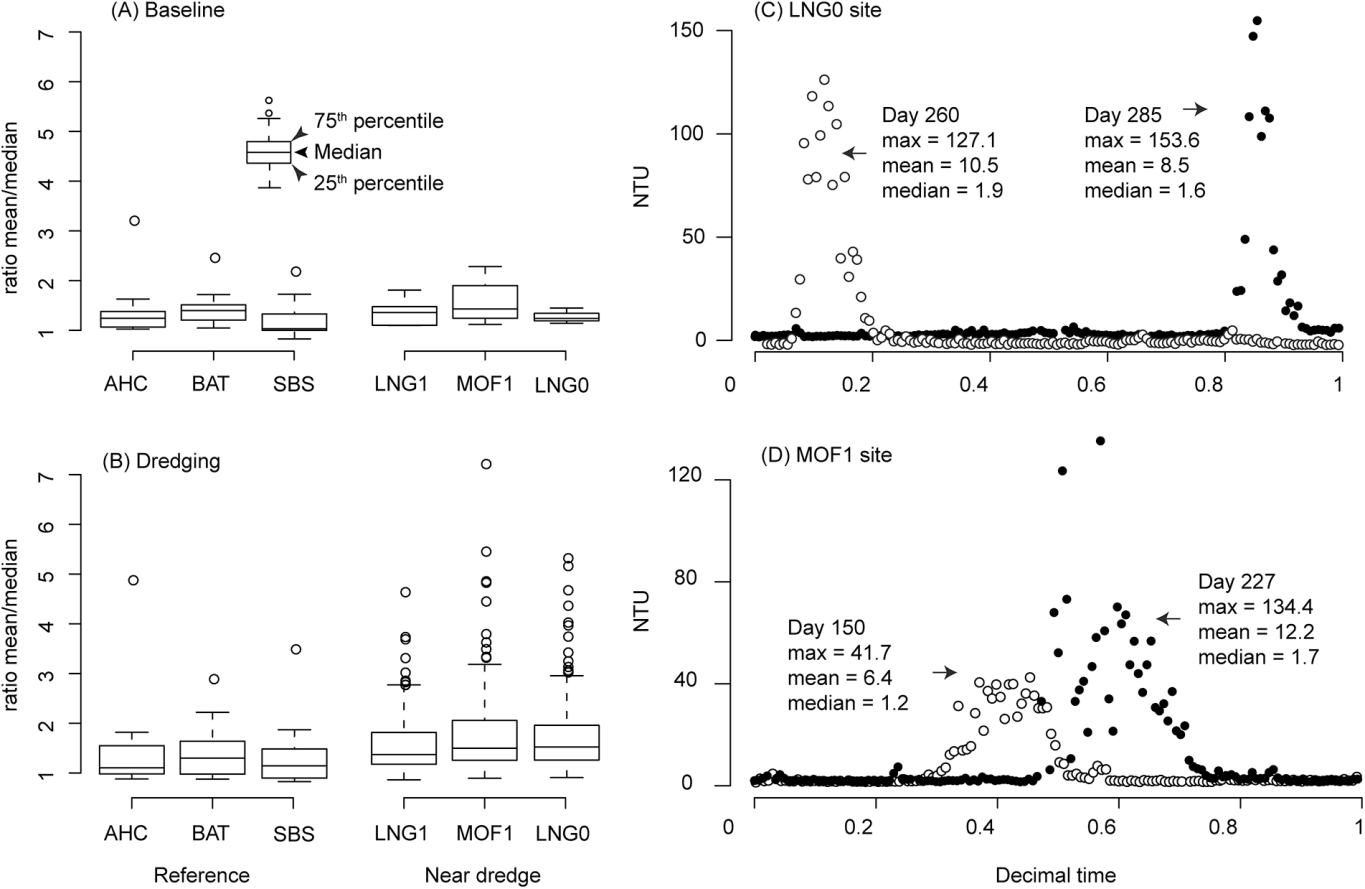
A comparison of mean versus median values as a statistical summary for daily turbidity (NTU) readings for selected near dredge and reference sites at Barrow Island. Boxplots of the ratio of the mean daily value versus the median daily value are shown during (A) the baseline period and (B) the dredging phase. The central bar of the box represents the median value, with the hinges indicating the first and third quartiles, and whiskers extending to the most extreme data point within 1.5 times this interquartile range. Only days where the maximum turbidity reading at any time throughout the day was greater than 20 NTU were included. This ratio was greater than 5 fold for four days from two sites, and the turbidity readings (NTU) of these days are plotted in C (LNG0) and D (MOF1).

Table 3 includes both median (50^th^ percentile) and mean values of turbidity over time–frames of one hour to 30 d. Data summarised as a mean gave greater values than when summarised via median in all time periods (with the ratio of mean to median usually greater than one). To further examine the relationship between median and mean, Fig 5 shows the ratio calculated for each day during the Barrow Island project for three reference sites (AHC, BAT, SBS) and three near dredge sites (LNG0, LNG1, MOF1) where there was a known high turbidity peak (>20 NTU, any time during the day). Mean daily turbidity values were over 5 times higher than the median for some days at sites impacted by dredging. For example, mean daily turbidity for LNG0 on day 285 was 8.5 NTU versus a median value of only 1.6 NTU, with the maximum turbidity observed for the day as high as 153.6 NTU (Fig 5C).

### Intensity, duration, frequency (IDF) analysis

Based on maximum daily values, the intensity (95^th^ percentile) of turbidity peaks was 11 times greater during the dredging period than the baseline at LNG1 (dredge impacted site) from the Barrow Island project, whereas reference sites (e.g., SBS) showed little change (Table 4). Although less pronounced, there was also an increase in the intensity of turbidity peaks for dredge impacted sites for the Cape Lambert and Burrup Peninsula projects with 2–3-fold increases in intensity (Table 4). In addition to an increase in intensity, both the duration and frequency of turbidity peaks also increased during dredging at dredge impacted sites (Table 4). The upper 95^th^ percentile of the duration of turbidity events ranged from 6.4 to 16 days at the dredging impacted sites during dredging, compared to only 1.9 to 3 days during baseline, representing a 1.8 to 5.3-fold increase (Table 4). The frequency of high turbidity events increased 2.8–3.4-fold across the three projects (Table 4).

**Table 4:** Intensity, duration and frequency (IDF) analysis of the seawater quality data at selected dredge-influenced site (Dredg.) and reference site (Ref.) for the Barrow Island, Cape Lambert and Burrup Peninsula dredging programs.

		Barrow Island		Cape Lambert		Burrup Peninsula	
	Period	Dredging (LNG1)	Reference (SBS)	Dredging (PWR)	Reference (DLI)	Dredging (SUP2)	Reference (WINI)
Daily							
	baseline	8	20	38	29	9	29
Intensity (I)	dredging	90	14	99	21	29	25
	change	11.0	0.7	2.6	0.7	3.1	0.9
	baseline	3.0	5.4	3.6	9.2	1.9	2.7
Duration (D)	dredging	16.0	2.0	6.4	6.6	7.2	2.9
	change	5.3	0.4	1.8	0.7	3.9	1.1
	baseline	12	6	8	6	12	12
Frequency (F)	dredging	34	6	28	5	34	9
	change	3.0	1.0	3.4	0.9	2.8	0.7
Hourly							
	baseline	4	10	19	10	6	14
Intensity (I)	dredging	30	7	42	9	11	9
	change	7.2	0.6	2.2	0.9	2.0	0.7
	baseline	0.3	0.8	0.4	0.8	0.2	0.2
Duration (D)	dredging	0.8	0.7	0.8	1.5	0.6	0.3
	change	2.7	1.0	2.3	1.9	2.8	1.4
	baseline	7	4	8	5	6	6
Frequency (F)	dredging	104	2	34	5	77	9
	change	13.5	0.5	4.4	1.0	12.6	1.5

The analysis was carried out separately at daily and hourly temporal scale. Intensity values represent the 95% percentile of turbidity for the site for each period. Duration values represent the 95^th^ percentile of the duration (days) of exceedance events (where exceedance events are defined as an event where the observed value exceeds the 95^th^ percentile (i.e. the intensity threshold) of the baseline state for that site). Frequency represents the number of times the duration of events exceeded the 95^th^ percentile of the duration of exceedance events for the baseline state for that site. Frequency has been normalised per year. ‘Change’ shows the value for the dredge period as a proportion of the baseline.

Results of the IDF analysis based on maximum hourly values were relatively consistent with those based on daily values, with 7.2, 2.2 and 2-fold increases in intensity; 2.7, 2.3 and 2.8-fold increases in duration; and 13.5, 4.4 and 12.6-fold increases in frequency at dredge impacted locations across the Barrow Island, Cape Lambert and Burrup Peninsula projects respectively. Over these two temporal scales there was little change occurring at representative reference sites, with both scales showing a 0.6–0.9-fold change (Table 4). There were, however, substantial differences in the actual values observed among the two temporal scales (Table 4). Hourly intensity values were significantly more variable and highly left skewed, thus 95% percentiles were much lower (11–41 NTU for hourly values versus 28–99 NTU for daily values), of shorter duration (0.6–0.8 days for hourly values versus 7.2–16.0 days for daily values) and far more frequent than their daily equivalents (34–104 exceedances for hourly values versus 28–34 exceedances for daily values, Table 4).

### Twilight periods

**Fig 6:**
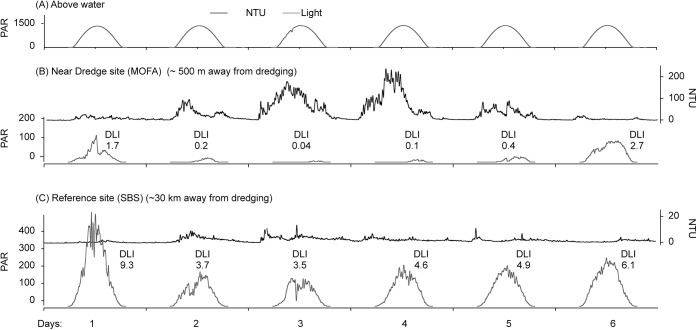
Turbidity (NTU) and PAR (μmol photons m^-2^ s^-1^) during the Barrow Island dredging project measured every 10 mins over a 6 day period in April 2011 from (A) a terrestrial light sensor located on Barrow Island (B) at 4.5 m depth at a site ~150 m from dredging, and (C) at 4.5 m depth at reference site ~30 km from dredging (see Fig 1). Numbers above the light profiles are the daily light integral (mol photons m^-2^ d^-1^) (see Material and Methods).

Irradiance levels across both the baseline and dredging periods were only consistently available for the Barrow Island project (Table 5). Higher turbidity values resulted in lower underwater light conditions, and a representative time series is shown graphically in Fig 6 for two sites at the same seawater depth (4.5 m) over a period of near uninterrupted sunshine (peaking at 1600 μmol photons m^-2^ s^-1^ at solar noon, Fig 6A). The site closest (~0.5 km) to the dredging experienced a turbidity event which peaked at ~200 NTU on days 3 and 4. Over the 6 days there were frequent low-light periods, and four days in a row where one half to one third of the daylight hours was in darkness. On day 3 of this sequence, instantaneous light levels peaked at only 6 μmol photons m^-2^ s^-1^ and the DLI was only 0.04 mol photons m^-2^ (Fig 6B). Over the same period at the reference site (>30 km away) the peak turbidity was more than one order of magnitude lower, light levels typically exceeded a maximum of ~200 μmol photons m^-2^ s^-1^ each day and the DLIs ranged from 3–9 mol photons m^-2^ (Fig 5C).

**Table 5:** The photosynthetically active radiation (PAR) daily light integral (DLI, mol photons m^-2^) percentile values for various running mean time periods for the Barrow Island dredging project.

		Percentile value (DLI, mol photons m^-2^)					
		0^th^ (min)	1^st^	5^th^	20^th^	50^th^	mean
Baseline/	1 d	0.1, 0.7	0.3, 1	1.1, 1.8	2.8, 3.5	4.3, 5.3	4.2, 5.2
reference		0–6.3	0–8.9	0.2–11	1.1–13	2.2–16	2.5–16
	14 d	1.5, 1.9	1.5, 2	2.1, 2.5	3.1, 3.5	4.2, 4.9	4.2, 4.9
		0.5–12	0.5–13	0.8–14	1.2–14	2.3–17	2.5–17
	30 d	2.1, 2.6	2.3, 2.7	2.4, 2.9	3.2, 3.7	4.3, 5	4.3, 5
		0.7–13	0.8–14	0.9–15	1.4–15	2.1–17	2.5–18
Near dredge	1 d	0, 0	0, 0	0.1, 0.1	0.8, 0.7	2.2, 1.9	2.1, 2.1
		0–0	0–0.1	0–0.5	0.2–1.7	0.7–3.8	2.1, 1–3.8
	14 d	0.3, 0.3	0.3, 0.4	0.5, 0.6	1.1, 1.2	2.3, 2.1	2.2, 2.2
		0.1–0.8	0.1–0.9	0.3–1.2	0.4–2.5	0.9–3.8	1–4
	30 d	0.4, 0.5	0.4, 0.5	0.6, 0.7	1, 1.3	2.4, 2.2	2.1, 2.2
		0.2–1.2	0.3–1.2	0.3–2.2	0.4–2.8	0.9–4.4	1.1–4.3

Percentiles were calculated separately for the baseline and reference site data (combined) and for near dredge sites (<2 km) during dredging. Shown are the median and mean and range across all relevant 0^th^ (minimum), 1^st^, 5^th^, 20^th^ and 50^th^ (median) percentiles for the one day, 14 d and 40 d running mean periods.

**Fig 7:**
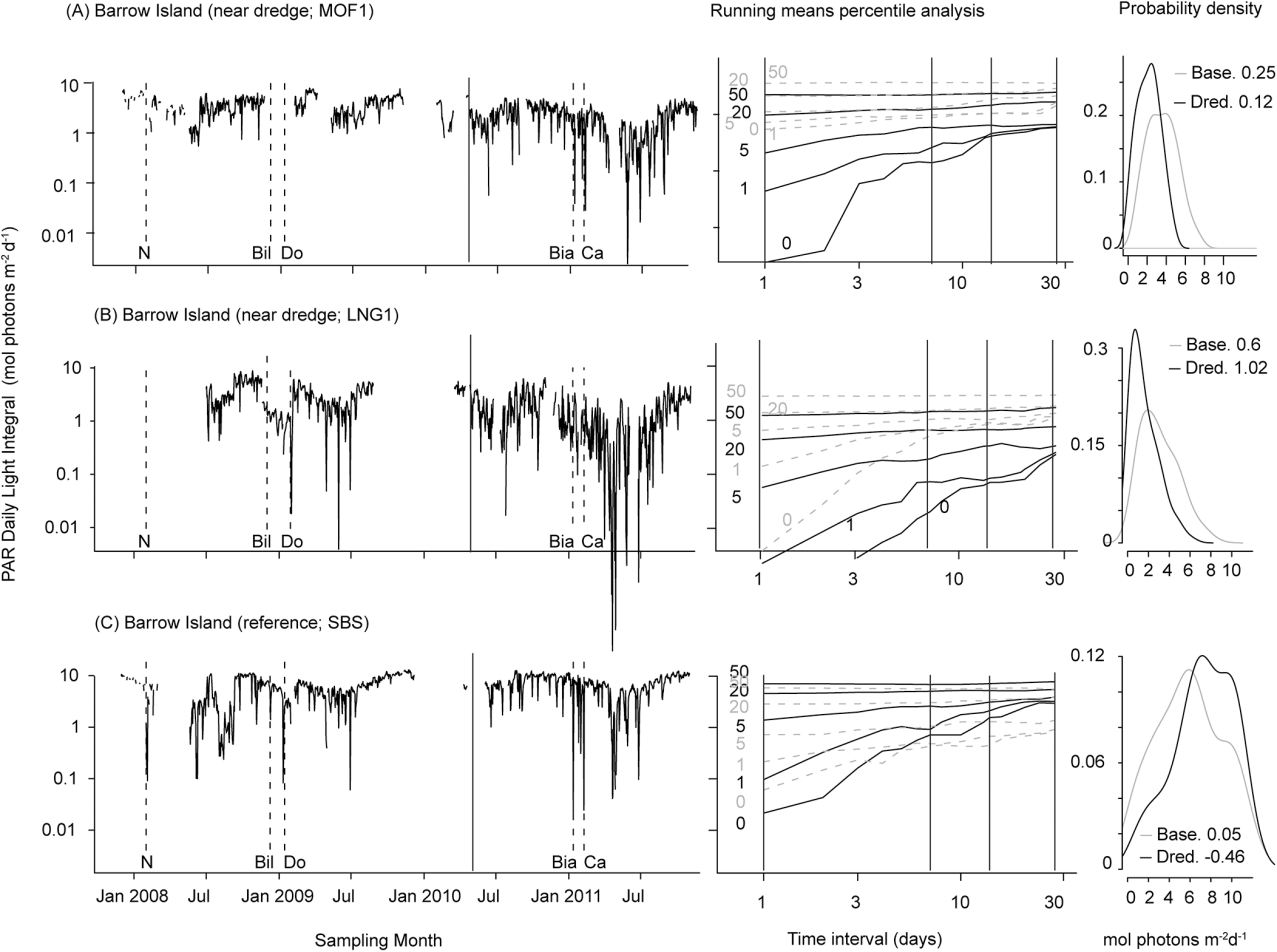
Total daily light integral (mol photons m^-2^ d^-1^, left panels) and probability density function (right panels) at two dredge impacted sites (MOF1 and LNG1, see Fig 1) and at SBS (reference site) during the Barrow Island project. Seawater depth at MOF1 and SBS were similar (~4.5 m) and at LNG1 was ~9 m. The red line on the left hand plots indicates the start of dredging for each project and dashed lines represent the timing of cyclone events that may have had the potential to cause substantial swell in this region (Puotinen, pers comm). Annotations at the base of the x-axis indicate each cyclone event, as follows: (a) Nicholas, max category 4, min distance 190 km; (b) Dominic, max category 2, min distance 20 km; (c) Bianca, max category 4, min distance 105 km; (d) Carlos, max category 3, minimum distance 0 km. Centre panels show the running means percentile analysis (50^th^, 20^th^, 5^th^, 1^st^ and 0^th^ (minimum)) PAR values, plotted as a function of the running mean time span from 1 to 30 days. Annotations under each axis indicate each cyclone event, as follows: Nicholas (N), category 4 minimum distance 190 km; Billy (Bil), category 3; Dominic (Do), category 2 minimum distance 20 km; Bianca (Bia) category 4, minimum distance 105 km; Carlos (Ca) minimum distance 0 km, category 3; Lua (Lu) category 4. Cyclone categories indicate the intensity (Australian Ranking Scale) of each cyclone at closest approach to the sites.

DLIs showed substantial drops episodically during the baseline period at both dredged and reference locations (Fig 7A and 7B). Both of these sites were at the same seawater depth (~4.5 m). However, both the frequency and intensity of drops in light availability were substantially greater during the dredging phase compared to the baseline, with values below 0.1 mol photons m^-2^ d^-1^ occurring regularly (Fig 7A) as indicated in the running means percentile analyses (centre panels in Fig 7, Table 5). As might be expected the trends resulting from dredging on the probability distribution of light were the inverse of those of NTU, with dredging increasing skewness due to an increasing frequency of low values and increasing kurtosis (Fig 7). Fig 7B shows a similar trend for a site 0.5 km from the dredging (site LNG1, see Fig 1) but where the loggers were located in deeper seawater (9 m). Over the dredging period ~5% of all values were below 0.1 mol photons m^-2^ d^-1^ and the site routinely experienced DLIs <0.04 mol photons m^-2^.

**Fig 8:**
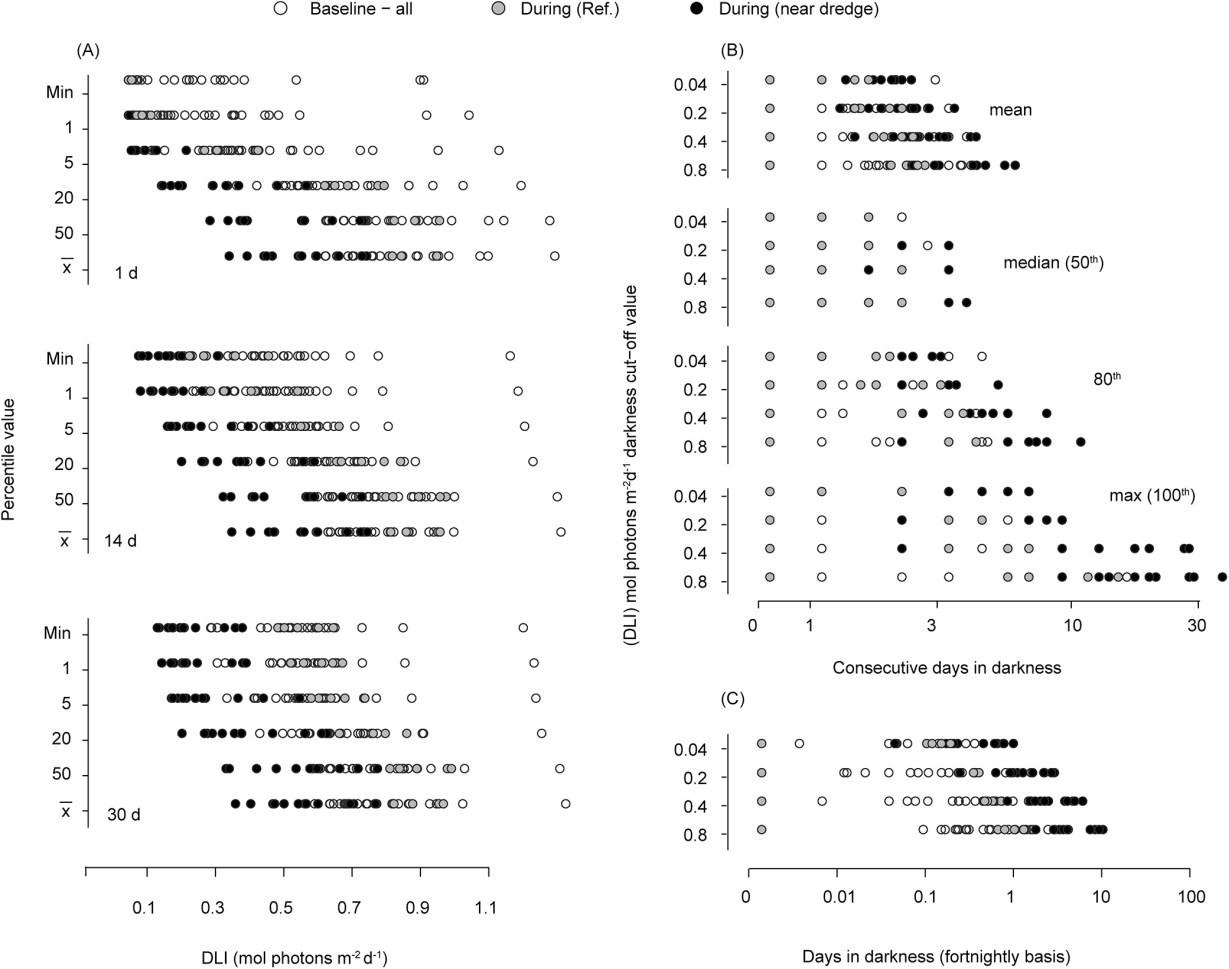
(A) Total photosynthetically active radiation (PAR) daily light integral (DLI, mol photons m^-2^) percentile values for running means calculated on time scales of 1, 14 and 30 days for all sites for the Barrow Island project. (B) Mean, median, 80^th^ percentile and maximum number of consecutive days in darkness and semi-darkness and (C) and mean fortnightly numbers of days for 4 different semi-darkness cut-off thresholds at all sites for the Barrow Island dredging project (1, 5, 10 and 20 μmol photons m^-2^ s^-1^; equivalent to DLI values of 0.04, 0.2, 0.4, and 0.8 mol photons m^-2^ d^-1^). White symbols represent percentiles for the baseline period (pre-dredging period), grey symbols represent reference sites during the dredging period and black symbols represent sites close to (<2 km) the dredging.

If low light is defined as an average instantaneous flux of 20 μmol photons m^-2^ s^-1^ (or approximately 1% of surface irradiance) for 12 h (equivalent to 0.8 mol photons m^-2^ d^-1^), dredge-influenced sites experienced more than 30 consecutive days in very reduced light levels (Fig 8A). If low light is defined as an average instantaneous flux of 5 μmol photons m^-2^ s^-1^ for 12 (~0.2 mol photons m^-2^ d^-1^) the worst case (maximum values) for near (<2 km) dredge sites during the dredging period was ~9 consecutive days, with the 80^th^ percentiles reaching 6 days and medians of ~3 days (Fig 8). This contrasts with the worst-case scenarios during baseline and at reference locations, which were ~5, ~4 and ~2 days respectively (maximum, 80^th^ and median, Fig 8).

Expressed as mean number of days per fortnight, dredge impacted sites were subjected to 2–7 (14–50%) days of very low light depending on the light cut-off values used (Fig 8B). Normalised per year, for one of the most light restricted definitions of low light (5 μmol photons m^-2^ s^-1^), the sites where the seawater quality was worst impacted sites can experience up to 70 days (20%) in effective low light and around 150 days (>40%) if less light restricted cut-off values are considered (data not shown).

## Discussion

During the dredging programs turbidity levels were highly variable, sometimes changing 2–3 orders of magnitude over the course of a day. Associated with these high turbidity events PAR levels also exhibit marked changes, frequently dropping to extremely low levels, creating daytime twilight and occasionally periods of darkness even in the shallow (4–5 m) reef environment. Under such highly variable conditions the choice of statistics is very important for summarising over time periods. Daily periods are often used to characterise seawater quality and using median values can miss quite substantial turbidity events if they only occur for a small part of the day period (cf Fig 5). Due to the ephemeral nature of the turbidity events care also needs to be taken when summarising data over longer time periods. For example, over the baseline period of the Barrow Island project, the average turbidity for the sites closest to the dredging was ~1.5 NTU slightly above the resolution of the nephelometers whilst during the dredging period it was 6.1 NTU. This statistic masks the fact that the sites were exposed to plumes for over 300 days during the dredging program ([35] and received maximum hourly average turbidity values sometimes exceeding hundreds of mg L^-1^ (200–400 NTU). These sites were within areas where coral mortality was permitted under regulatory conditions and where many corals suffered whole and/or partial mortality. Clearly the average was much less but the peaks much more, which is important as these peaks can have ecological consequences. Using an estimated turbidity to SSC conversion factor of 1:1.1 to1.6 during the dredging project, these sites received long term average SSCs just under the 10 mg L^-1^ threshold suggested by Rogers (1990) as indicative of reefs not subjected to stresses by humans, and used as a ‘rule-of-thumb’ for concern [13,36]. The frequently cited threshold value of 10 mg L^-1^ has little meaning without a temporal context i.e. *x* mg L^-1^ over *y* days.

Dredging effectively alters the overall probability distributions of fine temporal scale turbidity and light changes, increasing the frequency of extreme values and dampening the probability distribution by increasing the frequency of larger values, decreasing both skewness and kurtosis. When averaged across the entire baseline and dredging phases separately for the three Pilbara dredging projects turbidity values increased by 2–3 fold but when examined by the IDF analysis across baseline and dredging periods, dredging increased the intensity (magnitude) of turbidity peaks by over an order of magnitude, generated peaks that lasted five times longer than the baseline period, and may cause peaks to occur up to three times more frequently.

### Temporal scales analysis

The running means analysis of the turbidity data and light (Figs 3 and 6) provides an effective method for viewing seawater quality conditions at multiple different time intervals as well as considering the upper percentile values. Examining these upper values is important as they can have biological consequences ([37]) and the analyses are possible because of the frequent (typically every 10–30 mins) sampling undertaken during the seawater quality monitoring programs which has increased resolution for the upper percentiles and lowered the potential for bias [22]. A recent wavelet analysis of the turbidity data showed clear periodicities of turbidity in the three Pilbara datasets during both the baseline and dredge phases of the studies (Stark, unublished data) peaking semidiurnally associated with tides, diurnally associated with daily sea breezes and sometimes fortnightly associated with spring-neap cycles. The running means analyses were conducted to a period of up to 1 month, a time frame which accounts for the short term acute turbidity events (i.e. hours or a few days) as well and long term (chronic) periods (i.e. days and weeks) and encompasses the periodicities in the data. By then examining these running means periods using a range of percentile values (i.e. *P*_100_, *P*_99_, *P*_95_, *P*_80_, or *P*_50_) it is possible to describe the impacts that dredging has on seawater quality (relative to the baseline period or appropriate reference sites) simultaneously for both rare (upper percentiles values i.e. *P*_100-95_) and common (medium percentile values i.e. *P*_80_ and *P*_50_) turbidity events.

If the running means/percentile analysis is conducted using the baseline (i.e. pre-dredging) data it captures short term turbidity events, effectively characterising the natural turbidity regime at a location. These natural turbidity events are common in the marine environment and usually associated with wind-driven waves in the shallow reef environment [11–14,38]. Conducting the same analyses during the dredging operations (and under the influence of a dredging plume) captures the effect of dredging-related turbidity on top of the natural, background patterns. The analysis shows a clear shift in the running-mean-percentile profiles between baseline and dredging at impacted sites across the dredging programs.

Using the running means/percentile analysis, the *P*_100_ (i.e. maximum) for a given time interval typically decreased as the averaging period increased. That is, given the transitory nature of turbidity events, seawater quality conditions will usually become better over longer periods as conditions are likely to improve. As the percentile values decreased, the averages across broader time scales became more similar, with the *P*_80_ values showing relatively consistent values across the whole spectrum of time scales examined. While summary statistics for upper percentile values generally declined with increasing temporal scales, these patterns were not always smooth, and occasionally they increase as the time increment increased. One such inflection point can be seen around 14 days (see Fig 3C), although increases can occur across sites at a range of temporal scales (see Figs A-D in S2 File). This effect is due to the periodicity in turbidity discussed previously (Stark, unublished data), and in this case is certainly the 14.76 d spring-neap cycle [39], where turbidity is naturally higher during spring than neap tides associated with greater current velocities. As the averaging time intervals increases to beyond 2 weeks, it will begin to incorporate a second spring tide sequence and secondary peak, as opposed to only one during the shorter (7 d) time intervals.

### Daytime-twilight events

The second prominent and characteristic feature of the seawater quality conditions during the dredging programs were the low light caliginous, or ‘daytime twilight’ periods. Such conditions are well known, even for tropical environments, associated with wind and wave events [40,41]. Complete darkness was sometimes recorded during the baseline periods but occurred more frequently during the dredging program.

Defining light low as a DLI of 0.8 mol photons m^2^, or equivalent to 12 h of 20 μmol photons m^2^ s^-1^ or approximately 1% of surface illumination (the delineation between euphotic and dysphotic zones), benthic taxa may experience up to 30 continuous days, or up to 7 days per fortnight of low light conditions when under the influence of dredging plumes. Whilst natural caliginous periods can represent significant challenges to corals, they usually occur naturally over short time periods associated with the passage of storms. Loss of all daytime light can also occur during baseline periods but sometimes over extended periods during dredging. Defining complete darkness as a DLI of 0.04 mol photons m^2^ (or equivalent to 12 h of 1 μmol photons m^2^ s^1^) some sites remained in darkness for >5 consecutive days. Loss of all light for a whole day or loss of light for a significant portion of the day, followed by extreme low light for the remainder of the day (see Fig 5), may present physiological challenges to corals beyond those of a simple energy deficit. In sustained low light periods corals will expel their algal symbionts and this dissociation of the symbiosis causes coral to turn white (cf bleaching). Yonge and Nicholls [42] found that tropical corals will bleach within a few days of being placed in darkness. Kevin and Hudson [43] recorded a much longer-time frame for the temperate coral *Plesiastrea versipora* (Lamarck, 1816), suggesting adaptation to episodic periods of low light as is common in higher latitudes. The dissociation of the symbiosis has profound implications for corals as regaining the algal symbionts to stable-state densities takes several months [44]. Loss of the symbionts will prevent or reduce the ability of corals from regaining an energy deficit autotrophically between turbidity events. Understanding the effects these caliginous periods on the coral-algal symbiosis, and in particular whether full light exclusion as opposed to very low light levels induces bleaching, could be useful in developing light thresholds for dredging programs.

One of the objectives of this analysis was to provide a temporal analysis of seawater quality to allow the design of more realistic experiments examining the effects of sediments on tropical marine organisms (corals, seagrasses, sponges ascidians etc). The running means/percentile analysis described herein has provided a matrix of empirical data of seawater quality (turbidity and light levels) which when expressed as 100^th^ (maximum), 99^th^, 95^th^, 80^th^ and 50^th^ (median) percentiles over multiple time frames (hours to weeks) effectively captures the entire range of likely seawater quality conditions associated with dredging in a reefal environment. This provides a reference data set for designing future experiments (see [9]) and also for interpreting the results of previous studies.

### Seawater quality thresholds

A useful way of managing dredging programs is seawater quality monitoring i.e. measuring the key hazards or environmental ‘pressures’, which are capable of having adverse biological effects [2,45]. Given the ephemeral pattern of dredging related turbidity events, thresholds need really to be developed over telescoping time periods, from short term acute events through to longer term chronic time periods an approach that is increasingly adopted in Australia and Singapore. Episodic periods of poor seawater quality are often interspersed with periods of otherwise normal seawater quality, driven by meteorological and hydrological conditions (sea breezes and tidal patterns), and influenced by heterogeneity of the plumes. This may provide benthic communities with opportunities to partially or fully recover depending on the nature of the disturbance and this could be incorporated into thresholds.

This study has concentrated on changes in turbidity and light quantity associated with dredging and yet these are only some of the key cause–effect pathways of risk associated with turbidity generation in shallow tropical marine environments. Changes in light quality, and especially sediment deposition have not been considered here. Deposition rates that exceed the natural clearing ability of corals can result in sediment smothering the tissues [46]. Once this has occurred solute (gas) exchange and light availability will be very limited, and the corals’ health will become un-coupled or unrelated to changes in SSC and light in the overlying seawater column (but see [47]). Relating coral health to seawater quality during dredging program requires knowledge of all causes of mortality and especially the potential influence of sediment deposition and incorrect identification of the relevant route(s) of exposure could be very misleading [48].

### Dredge material placement sites

Ocean disposal of sediment at dredge material placement sites (spoil grounds) is another potentially significant turbidity-generating event associated with dredging. Plumes can be generated as the sediments are released over the disposal grounds and fine material in the water column can migrate to nearby habitats. In the longer term, this material and any fine material within the disposal site could be subsequently mobilized by storms and dispersed further. The extent to which mobilization and movement from the disposal ground occurs is determined by whether the site is located within a sediment transport pathway with a high or low throughput i.e. is dispersive or retentive site, and in turn dependent on bathymetry and hydrodynamics and coastline features (bays compared to promontories)[49]. Currently the long term fate and effects of ocean disposal is a significant issue on the Great Barrier Reef and the disposal of capital dredge material in the Great Barrier Reef Marine Park has recently been banned ([49]). In two of the three case studies described here there was no monitoring around the dredge material placement sites but some monitoring occurred at the Barrow Island sites with sensors placed ~1 km north and south of the 9 km^2^ spoil ground. Turbidity associated with sediment disposal at the placement sites was quite low as compared to extraction at the site of dredging (see **S1 File** and Fig B in S2 File) and consistent with Moderate Resolution Imaging Spectroradiometer (MODIS) satellite image analysis of the dredge plume boundaries during the Barrow Island project [35].

In conclusion, the data from the recent large-scale capital projects in Australia’s Pilbara region have produced very detailed information on the changes that can occur in seawater quality during dredging in coral reef environments. Characteristic features are the highly variable and transitory nature of the turbidity events and the pronounced increase in the intensity, duration and frequency of turbidity compared to natural background events. Associated with the turbidity are profound changes in submarine light fields, with frequent and often extended low light caliginous or ‘twilight’ periods and sometimes loss of all light. The choice of summary statistic and analysis periods is very important for describing such highly variable data as median values or longer term averaging periods can hide significant events which could have ecological consequences. The broad spatial and temporal coverage together with the statistical approaches and methods of analysis used here have provided information that is important for contextualising seawater quality information in future dredging programs. The same information can be used in manipulative studies examining the effects of dredging on tropical marine organisms using environmentally realistic and relevant exposure conditions. Collectively this information could contribute to the development of seawater quality thresholds for dredging projects and ultimately improve the ability to predict and manage the impact of future projects.

## Supporting Information

S1 File Sampling and site information for all seawater quality monitoring sites. Detailed site information, including depth below LAT and distance (km) from dredging activity (where relevant) during the three Pilbara (Western Australia) dredging projects. The number of valid sample days for NTU and light are shown for the baseline and dredging periods, as are the mean values at NTU and light (µmol photons m^-2^ s^-1^) across all samples for each period.
(DOCX)

S2 File Maximum instantaneous daily turbidity (NTU) or light (µmol photons m^-2^ s^-1^) during the baseline period (before dredging) or during the dredging program (left), probability density curves (mid) and running mean quantile plot (right) for the Burrup Peninsula project (Figure A), Barrow Island project (Figure B and D) and Cape Lambert project (Figure C). Running mean quantile plots show the 100^th^ (maximum), 99^th^ and 95^th^ and 80^th^ percentile of running periods from 1 h to 21 d before (dashed lines) and during (solid lines) the dredging program. Data are only shown for near dredge sites (<2 km) and those site considered reference sites. Vertical red lines on the left-hand time series plots show cyclone events that may impact sites. Time series, probability density, and running means for all sites during the Burrup Peninsula Project (Figure A), Barrow Island project (Figure B), and Cape Lambert Project (Figure C). Fig. A. Burrup Peninsula project. NTU data. Time series, probability density, and running means for all sites. Fig. B. Barrow Island project. NTU data. Time series, probability density, and running means for all sites. Fig. C. Cape Lambert project. NTU data. Time series, probability density, and running means for all sites. Fig. D. Barrow Island project. Light data. Time series, probability density, and running means for all sites.
(DOCX)

## References

[1] Rogers CS (1990) Responses of coral reefs and reef organisms to sedimentation. Marine Ecology Progress Series 62: 185–202.

[2] Foster T, Corcoran E, Erftemeijer P, Fletcher C, Peirs K, et al. (2010) Dredging and port construction around coral reefs. PIANC Environmental Commission, Report No 108.

[3] Fabricius KE (2005) Effects of terrestrial runoff on the ecology of corals and coral reefs: review and synthesis. Marine Pollution Bulletin 50: 125–146. 15737355 10.1016/j.marpolbul.2004.11.028

[4] Erftemeijer PL, Riegl B, Hoeksema BW, Todd PA (2012) Environmental impacts of dredging and other sediment disturbances on corals: a review. Marine Pollution Bulletin 64: 1737–1765. 10.1016/j.marpolbul.2012.05.008 22682583

[5] Bak RPM (1978) Lethal and sublethal effects of dredging on reef coral. Marine Pollution Bulletin 9: 14–16.

[6] Johannes RE (1970) How to kill a coral reef. Marine Pollution Bulletin 1: 186–187.

[7] Stoddart J, Anstee S (2004) Water quality, plume modelling and tracking before and during dredging in Mermaid Sound, Dampier, Western Australia In: Stoddart JA, Stoddart SE, editors. Corals of the Dampier Harbour: their survival and reproduction during the dredging programs of 2004: MScience Pty Ltd, University of Western Australia, Perth, Western Australia pp. 13–33.

[8] Trimarchi S, Keane J (2007) Port of Hay Point Apron Areas and Departure Path Capital Dredging Project Environmental Review, EcoPorts Monograph Series No. 24, Ports Corporation of Queensland, Brisbane.

[9] Norris RH, Webb JA, Nichols SJ, Stewardson MJ, Harrison ET (2011) Analyzing cause and effect in environmental assessments: using weighted evidence from the literature. Freshwater Science 31: 5–21.

[10] Macdonald RK, Ridd PV, Whinney JC, Larcombe P, Neil DT (2013) Towards environmental management of water turbidity within open coastal waters of the Great Barrier Reef. Marine Pollution Bulletin 74: 82–94. 10.1016/j.marpolbul.2013.07.026 23948091

[11] Jing L, Ridd PV (1996) Wave-current bottom shear stresses and sediment resuspension in Cleveland Bay, Australia. Coastal Engineering 29: 169–186.

[12] Larcombe P, Costen A, Woolfe KJ (2001) The hydrodynamic and sedimentary setting of nearshore coral reefs, central Great Barrier Reef shelf, Australia: Paluma Shoals, a case study. Sedimentology 48: 811–835.

[13] Ogston AS, Storlazzi CD, Field ME, Presto MK (2004) Sediment resuspension and transport patterns on a fringing reef flat, Molokai, Hawaii. Coral Reefs 23: 559–569.

[14] Verspecht F, Pattiaratchi C (2010) On the significance of wind event frequency for particulate resuspension and light attenuation in coastal waters. Continental Shelf Research 30: 1971–1982.

[15] Fabricius K, Logan M, Weeks S, Brodie J (2014) The effects of river run-off on water clarity across the central Great Barrier Reef. Marine Pollution Bulletin 84: 191–200. 10.1016/j.marpolbul.2014.05.012 24863415

[16] Fabricius KE, De’ath G, Humphrey C, Zagorskis I, Schaffelke B (2013) Intra-annual variation in turbidity in response to terrestrial runoff on near-shore coral reefs of the Great Barrier Reef. Estuarine, Coastal and Shelf Science 116: 57–65.

[17] Thompson A, Schroeder T, Brando VE, Schaffelke B (2014) Coral community responses to declining water quality: Whitsunday Islands, Great Barrier Reef, Australia. Coral Reefs: 1–16.

[18] Larcombe P, Ridd P, Prytz A, Wilson B (1995) Factors controlling suspended sediment on inner-shelf coral reefs, Townsville, Australia. Coral Reefs 14: 163–171.

[19] Orpin A, Ridd P, Thomas S, Anthony K, Marshall P, et al (2004) Natural turbidity variability and weather forecasts in risk management of anthropogenic sediment discharge near sensitive environments. Marine Pollution Bulletin 49: 602–612. 15476839 10.1016/j.marpolbul.2004.03.020

[20] Cooper TF, Ridd PV, Ulstrup KE, Humphrey C, Slivkoff M, et al (2008) Temporal dynamics in coral bioindicators for water quality on coastal coral reefs of the Great Barrier Reef. Marine and Freshwater Research 59: 703–716.

[21] Orpin AR, Ridd PV (2012) Exposure of inshore corals to suspended sediments due to wave-resuspension and river plumes in the central Great Barrier Reef: A reappraisal. Continental Shelf Research 47: 55–67.

[22] Falkenberg LJ, Styan CA (2014) Too much data is never enough: A review of the mismatch between scales of water quality data collection and reporting from recent marine dredging programmes. Ecological Indicators 45: 529–537.

[23] Zimmermann NE, Yoccoz NG, Edwards TC, Meier ES, Thuiller W, et al (2009) Climatic extremes improve predictions of spatial patterns of tree species. Proceedings of the National Academy of Sciences of the United States of America 106: 19723–19728. 10.1073/pnas.0901643106 19897732 PMC2780931

[24] Gaines SD, Denny MW (1993) The Largest, Smallest, Highest, Lowest, Longest, and Shortest: Extremes in Ecology. Ecology 74: 1677–1692.

[25] Hanley JR (2011) Environmental monitoring programs on recent capital dredging projects in the Pilbara (2003–10): a review. Australian Petroleum Production & Exploration Association (APPEA) 51 273–294.

[26] EPA (2011) Environmental Assessment Guidleine for Marine Dredging Programs EAG7. Environmental Protection Authority (EPA), Perth, Western Australia pp. 36.

[27] Davies-Colley R, Smith D (2001) Turbidity, suspended sediment and water quality: a review. Journal of the American Water Works Association 37: 1085–1101.

[28] Wood SN (2006) Generalized Additive Models: an introduction with R. Boca Raton, FL: CRC Press. 410 p.

[29] R Core Team (2014) R: A language and environment for statistical computing R Foundation for Statistical Computing, Vienna, Austria Available: http://www.R-project.org/.

[30] McArthur C, Ferry R, Proni J (2002) Development of guidelines for dredged material disposal based on abioytic determinants of coral reef community structure. Proceedings of the Third Specialty Conference on Dredging and Dredged Material Disposal Coasts, Oceans, Ports, and Rivers Institute (COPRI) of ASCE. Orlando, FL USA. 1–15.

[31] Newcombe CP, MacDonald DD (1991) Effects of suspended sediments on aquatic ecosystems. North American Journal of Fisheries Management 11: 72–82.

[32] Wilber DH, Clarke DG (2001) Biological effects of suspended sediments: a review of suspended sediment impacts on fish and shellfish with relation to dredging activities in estuaries. North American Journal of Fisheries Management 21: 855–875.

[33] Zeileis A, Grothendieck G (2005) zoo: S3 Infrastructure for Regular and Irregular Time Series. Journal of Statistical Software 14: 1–27.

[34] Tuszynski J (2013) caTools: Tools: moving window statistics, GIF, Base64, ROC AUC, etc. R package version 116. Available: http://CRANR-projectorg/package=caTools.

[35] Evans RD, Murray KL, Field SN, Moore JA, Shedrawi G, et al (2012) Digitise this! A quick and easy remote sensing method to monitor the daily extent of dredge plumes. PLoS ONE 7: e51668 10.1371/journal.pone.0051668 23240055 PMC3519868

[36] Ogston AS, Field ME (2010) Predictions of turbidity due to enhanced sediment resuspension resulting from sea-level rise on a fringing coral reef: evidence from Molokai, Hawaii. Journal of Coastal Research 26: 1027–1037.

[37] De'ath G, Fabricius K (2008) Water quality of the Great Barrier Reef: distributions, effects on reef biota and trigger values for the protection of ecosystem health: Great Barrier Reef Marine Park Authority.

[38] Lawrence D, Dagg MJ, Liu H, Cummings SR, Ortner PB, et al (2004) Wind events and benthic-pelagic coupling in a shallow subtropical bay in Florida. Marine Ecology Progress Series 266: 1–13.

[39] Kvale EP (2006) The origin of neap–spring tidal cycles. Marine Geology 235: 5–18.

[40] Anthony K, Larcombe P (2000) Coral reefs in turbid waters: sediment-induced stresses in corals and likely mechanisms of adaptation. Proceedings of the 9^th^ International Coral Reef Symposium Bali, Indonesia 1: 239–244.

[41] Jones RJ (2008) Coral bleaching, bleaching-induced mortality, and the adaptive significance of the bleaching response. Marine Biology 154: 65–80.

[42] Yonge CM, Nicholls A (1931) Studies on the physiology of corals. V The effects of starvation in light and in darkness on the relationship between corals and zooxanthellae. Great Barrier Reef Expedition 1928–29, Scientific Reports British Museum (Natural History) London (UK) 13–57 British Museum 1.

[43] Kevin KM, Hudson RCL (1979) The rôle of zooxanthellae in the hermatypic coral Plesiastrea urvillei (Milne Edwards and Haime) From cold waters. Journal of Experimental Marine Biology and Ecology 36: 157–170.

[44] Jones RJ, Yellowlees DY (1997) Algal (= zooxanthellae) regulation and control in hard corals. Philosophical Transactions of the Royal Society of London Series B: Biological Science 352: 457–468.

[45] CEDA (2015) Environmental monitoring procedures Information paper. Central Dredging Association (CEDA) Delft The Netherlands (http://www.dredging.org/media/): 23 pp.

[46] Stafford-Smith M, Ormond R (1992) Sediment-rejection mechanisms of 42 species of Australian scleractinian corals. Marine & Freshwater Research 43: 683–705.

[47] Junjie RK, Browne NK, Erftemeijer PLA, Todd PA (2014) Impacts of Sediments on Coral Energetics: Partitioning the Effects of Turbidity and Settling Particles. PLoS ONE 9: e107195 10.1371/journal.pone.0107195 25197883 PMC4157877

[48] Harris CA, Scott AP, Johnson AC, Panter GH, Sheahan D, et al (2014) Principles of Sound Ecotoxicology. Environmental Science & Technology 48: 3100–3111. 24512103 10.1021/es4047507

[49] McCook L, Schaffelke B, Apte A, Brinkman R, Brodie J, et al (2015) Synthesis of current knowledge of the biophysical impacts of dredging and disposal on the Great Barrier Reef: report of an independent panel of experts Great Barrier Reef Marine Park Authority, Townsville.

