# Supplementary material for: Temporal Patterns in Seawater Quality from Dredging in Tropical Environments
Source: PLoS One. 2015 Oct 7;10(10):e0137112. doi: 10.1371/journal.pone.0137112 (PMC4596475; doi:10.1371/journal.pone.0137112)
Supplement: S1 File — Detailed site information, including depth below LAT and distance (km) from dredging activity (where relevant) during the three Pilbara (Western Australia) dredging projects. The number of valid sample days for NTU and light are shown for the baseline and dredging periods, as are the mean values at NTU and light (µmol photons m-2 s-1) across all samples for each period. (DOCX) [file pone.0137112.s001.docx]

| Site information | | | | Number of days sampled | | | | Mean NTU and PAR | | | |
| --- | --- | --- | --- | --- | --- | --- | --- | --- | --- | --- | --- |
|  |  |  |  | Baseline | | Dredging | | Baseline | | Dredging | |
| Site | | Dist. | Dep. | NTU | Light | NTU | Light | NTU | Light | NTU | Light |
| Burrup Peninsula (MS757) | | | | | | | | | | | |
| Near | |  |  |  |  |  |  |  |  |  |  |
| CHC4 | | 0.4 | 2.0 | 96 | 114 | 49 |  | 1.9 | 27.3 | 35.4 |  |
| DPAN | | 0.6 |  | 5 | 4 | 47 | 82 | 1.5 | 33.8 | 31.3 | 13.2 |
| HOLD | | 0.3 |  | 5 | 4 | 83 | 82 | 0.1 | 141.9 | 28.2 |  |
| SUP2 | | 1.8 | 2.0 |  |  | 866 |  |  |  | 4.5 |  |
| Far | |  |  |  |  |  |  |  |  |  |  |
| FFP1 | | 13.8 | 2.5 | 16 |  | 984 |  | 1.8 |  | 2.0 |  |
| FFP2 | | 14.8 |  |  |  |  |  |  |  |  |  |
| INTI | | 11.8 |  |  |  |  |  |  |  |  |  |
| LEGD | | 28.6 | 9.0 | 11 |  | 871 |  | 0.4 |  | 0.6 |  |
| MAL2 | | 13.7 |  |  |  | 902 |  |  |  | 2.1 |  |
| MALI | | 9.9 | 3.5 |  |  | 923 |  |  |  | 1.3 |  |
| MIDI | | 12.0 | 3.0 |  |  | 933 |  |  |  | 3.3 |  |
| MIDR | | 15.9 | 4.0 |  |  | 860 |  |  |  | 1.3 |  |
| WINI | | 16.5 | 1.5 | 106 | 115 | 907 |  | 3.1 | 72.7 | 3.2 |  |
| WLI1 | | 14.6 |  |  |  |  |  |  |  |  |  |
| Barrow Island (MS800) | | | | | | | | | | | |
| Near |  | |  |  |  |  |  |  |  |  |  |
| LNG0 | 0.2 | | 9.0 | 457 | 476 | 479 | 482 | 1.2 | 60.6 | 6.1 | 29.7 |
| LNG1 | 0.5 | | 10.0 | 629 | 450 | 481 | 477 | 1.3 | 60.4 | 5.3 | 32.8 |
| LNG2 | 1.0 | | 7.0 | 632 | 636 | 510 | 442 | 1.0 | 76.3 | 3.8 | 50.9 |
| LNGA | 0.3 | | 10.0 | 117 | 113 | 488 | 471 | 1.3 | 51.7 | 6.5 | 19.7 |
| LNGB | 0.7 | | 8.5 | 94 | 115 | 501 | 481 | 2.1 | 57.9 | 5.5 | 30.5 |
| LNGC | 1.4 | | 8.5 | 249 | 241 | 486 | 425 | 1.0 | 59.1 | 4.8 | 24.5 |
| LOW | 1.9 | | 3.0 | 692 | 75 | 459 |  | 1.4 | 180.0 | 1.4 |  |
| LOW1 | 1.6 | | 8.0 | 226 | 173 | 449 | 524 | 1.1 | 78.3 | 1.3 | 79.6 |
| MOF1 | 0.8 | | 7.0 | 678 | 562 | 505 | 512 | 1.3 | 69.8 | 5.0 | 42.9 |
| MOF3 | 1.5 | | 6.0 | 657 | 549 | 487 | 488 | 1.4 | 95.4 | 3.2 | 76.2 |
| MOFA | 0.6 | | 6.0 | 144 | 137 | 471 | 455 | 1.8 | 65.2 | 7.1 | 61.5 |
| MOFB | 1.0 | | 6.0 | 130 | 211 | 521 | 488 | 1.5 | 71.9 | 4.1 | 43.6 |
| MOFC | 0.7 | | 6.0 | 121 | 154 | 460 | 472 | 2.1 | 71.6 | 6.6 | 44.7 |
| Far |  | |  |  |  |  |  |  |  |  |  |
| AHC | 32.8 | | 8.5 | 668 | 612 | 500 | 548 | 1.6 | 68.7 | 1.3 | 95.5 |
| REFN | 28.0 | | 4.5 | 91 | 93 | 370 | 426 | 0.8 | 64.8 | 1.5 | 103.9 |
| REFS | 23.6 | | 4.5 | 146 | 144 | 374 | 428 | 2.2 | 136.9 | 1.5 | 137.9 |
| SBS | 30.0 | | 4.5 | 599 | 605 | 454 | 502 | 2.6 | 116.1 | 1.6 | 138.4 |
| Spoil |  | |  |  |  |  |  |  |  |  |  |
| LONE | 0.7-5.0 | | 8.5 | 706 | 735 | 485 | 532 | 1.0 | 49.0 | 1.5 | 64.1 |
| DSGS | 0.7-5.0 | | 14 | 125 | 100 | 387 | 484 | 0.7 | 51.6 | 1.7 | 59.2 |
| Cape Lambert (MS 848) | | | | | | | | | | | |
| Near | |  |  |  |  |  |  |  |  |  |  |
| BTR | | 1.8 | 12.0 | 467 | 85 | 646 | 649 | 2.5 | 239.9 | 2.8 | 144.0 |
| PWR | | 1.2 | 6.0 | 399 | 91 | 629 | 686 | 3.7 | 438.2 | 8.9 | 225.6 |
| Far | |  |  |  |  |  |  |  |  |  |  |
| DLI | | 17.7 | 9.0 | 425 | 279 | 675 | 609 | 2.1 | 200.0 | 2.1 | 218.0 |
| DOI | | 35.4 | 7.0 | 13 | 12 | 686 | 607 | 1.2 | 335.3 | 2.9 | 251.6 |
| HAT | | 13.9 | 4.0 | 389 | 136 | 661 | 649 | 3.1 | 252.1 | 4.6 | 230.7 |
| SMSB | | 5.6 | 4.0 | 481 | 91 | 698 | 592 | 3.6 | 402.9 | 5.3 | 201.6 |

Data from a total of 83 sites were collated from the three major Pilbara dredging programs (32 sites from the Burrup Peninsula project, 36 sites from the Barrow Island project and 15 sites from the Cape Lambert project). Although there were 83 sites in total, for the purpose of the present analysis data was only used for sites that were in the immediate vicinity of dredging activity (near dredge, <2 km away) and those sites that were deemed appropriate reference sites for each project according to their approvals documents (far sites). Data collected at each site varied, as did the sampling effort during both baseline and dredging periods. For the Barrow Island project, two sites were situated near to the spoil ground (between 0.7 and 5.0 km, depending on which part of the spoil ground is used).
