# Supplementary material for: Temporal Patterns in Seawater Quality from Dredging in Tropical Environments
Source: PLoS One. 2015 Oct 7;10(10):e0137112. doi: 10.1371/journal.pone.0137112 (PMC4596475; doi:10.1371/journal.pone.0137112)
Supplement: S2 File — Running mean quantile plots show the 100th (maximum), 99th and 95th and 80th percentile of running periods from 1 h to 21 d before (dashed lines) and during (solid lines) the dredging program. Data are only shown for near dredge sites (<2 km) and those site considered reference sites. Vertical red lines on the left-hand time series plots show cyclone events that may impact sites. Time series, probability density, and running means for all sites during the Burrup Peninsula Project (Figure A), Barrow Island project (Figure B), and Cape Lambert Project (Figure C). Fig. A. Burrup Peninsula project. NTU data. Time series, probability density, and running means for all sites. Fig. B. Barrow Island project. NTU data. Time series, probability density, and running means for all sites. Fig. C. Cape Lambert project. NTU data. Time series, probability density, and running means for all sites. Fig. D. Barrow Island project. Light data. Time series, probability density, and running means for all sites. (DOCX) [file pone.0137112.s002.docx]

| **S2 Fig. A. Burrup Peninsula project**. | | | |
| --- | --- | --- | --- |
| CHC4 (near) |  | |  |
|  |  | |  |
| DPAN (near) |  | |  |
|  |  | |  |
| FFP1 (far) |  | |  |
|  |  | |  |
| HOLD (near) |  | |  |
|  |  | |  |
| LEGD (far) |  | |  |
|  |  | |  |
| NTU versus month | Probability function v NTU | NTU v time (days) | |

| MAL2 (far) |  |  |
| --- | --- | --- |
|  | **** |  |
| MALI (far) |  |  |
|  |  |  |
| MIDI (far) |  |  |
|  |  |  |
| MIDR (far) |  |  |
|  |  |  |
| SUP2 (near) |  |  |
|  |  |  |
| WINI (far) |  |  |
|  |  |  |
| NTU versus month | Probability function v NTU | NTU v time (days) |

| **S2 Fig. B. Barrow Island Project** | | | |
| --- | --- | --- | --- |
| AHC (far) |  | |  |
|  |  | |  |
| ELS (far) |  | |  |
|  |  | |  |
| REFN (far) |  | |  |
|  |  | |  |
| REFS (far) |  | |  |
|  |  | |  |
| SBS (far) |  | |  |
|  |  | |  |
| NTU versus month | Probability function v NTU | NTU v time (days) | |

| LOW (near) |  | |  |
| --- | --- | --- | --- |
|  |  | |  |
| LOW1 (near) |  | |  |
|  |  | |  |
| LNGA (near) |  | |  |
|  |  | |  |
| LNG0 (near) |  | |  |
|  |  | |  |
| LNG1 (near) |  | |  |
|  |  | |  |
| LNG2 (near) |  | |  |
|  |  | |  |
| NTU versus month | Probability function v NTU | NTU v time (days) | |

| LNGB (near) |  | |  |
| --- | --- | --- | --- |
|  |  | |  |
| LNGC (near) |  | |  |
|  |  | |  |
| MOF1 (near) |  | |  |
|  |  | |  |
| MOF3 (near) |  | |  |
|  |  | |  |
| MOFA (near) |  | |  |
|  |  | |  |
| MOFB (near) |  | |  |
|  |  | |  |
| NTU versus month | Probability function v NTU | NTU v time (days) | |

| MOFC (near) |  | |  |
| --- | --- | --- | --- |
|  |  | |  |
| DSGS (spoil) |  | |  |
|  |  | |  |
| NTU versus month | Probability function v NTU | NTU v time (days) | |
| LONE (spoil) |  | |  |
|  |  | |  |
| NTU versus month | Probability function v NTU | NTU v time (days) | |

| **S2 Fig C. Cape Lambert project.** | | | |
| --- | --- | --- | --- |
| BTR (near) |  | |  |
|  |  | |  |
| DLI (far) |  | |  |
|  |  | |  |
| DOI (far) |  | |  |
|  |  | |  |
| HAT (far) |  | |  |
|  |  | |  |
| PWR (near) |  | |  |
|  |  | |  |
| NTU versus month | Probability function v NTU | NTU v time (days) | |

| **S2 Fig D. Barrow Island project.** | | | | |
| --- | --- | --- | --- | --- |
| AHC (far) | |  | |  |
|  | |  | |  |
| ELS (far) | |  | |  |
|  | |  | |  |
| REFN (far) |  | | |  |
|  |  | | |  |
| REFS (far) |  | | |  |
|  |  | | |  |
| SBS (far) |  | | |  |
|  |  | | |  |
| NTU versus month | Probability function v NTU | | NTU v time (days) | |

| LOW (near) |  |  |
| --- | --- | --- |
|  |  |  |
| LOW1 (near) |  |  |
|  |  |  |
| LNGA (near) |  |  |
|  |  |  |
| LNG0 (near) |  |  |
|  |  |  |
| LNG1 (near) |  |  |
|  |  |  |
| LNG2 (near) |  |  |
|  |  |  |
| NTU versus month | Probability function v NTU | NTU v time (days) |

| LNGB (near) |  | | |  |
| --- | --- | --- | --- | --- |
|  |  | | |  |
| LNGC (near) |  | | |  |
|  |  | | |  |
| MOF1 (near) |  | | |  |
|  |  | | |  |
| MOF3 (near) |  | | |  |
|  |  | | |  |
| MOFA (near) | |  | |  |
|  | |  | |  |
| MOFB (near) | | |  |  |
|  | | |  |  |
| NTU versus month | | | Probability function v NTU | NTU v time (days) |

| MOFC (near) | | |  |  |
| --- | --- | --- | --- | --- |
|  | | |  |  |
| DSGS (spoil) | |  | |  |
|  | |  | |  |
| LONE (spoil) | |  | |  |
|  | |  | |  |
| Light versus month | Probability density function v light | | | Light v time (days) |
